# Differential Expression of *ACAN*, *COL1A1*, and *COL2A1* in Cartilage and Synovium During Osteoarthritis Progression: An Observational Pilot Study Supported by Preliminary Bioinformatic Analysis

**DOI:** 10.3390/jcm15207977

**Published:** 2026-10-15

**Authors:** Karolina Kopacz, Anna Litwin, Kamila Baran, Jakub Mateusz Kryczka, Aleksandra Czechowska, Gianluca Padula, Krzysztof Nowak, Starska-Kowarska Katarzyna, Joanna Boncela, Marcin Domżalski, Ewa Brzeziańska-Lasota

**Affiliations:** 1 Department of Physiology, Pathophysiology and Clinical Immunology, Department of Clinical Physiology, Medical University of Lodz, 90-752 Lodz, Poland; aleksandra.czechowska@umed.lodz.pl (A.C.); katarzyna.starska@umed.lodz.pl (S.-K.K.); 2 Faculty of Medical Science, Warsaw Medical Academy, 01-793 Warsaw, Poland; 3 Department of Biomedicine and Genetics, Biology and Medical Microbiology, Medical University of Lodz, 92-215 Lodz, Poland; annalitwin92@gmail.com (A.L.); kamila.baran@umed.lodz.pl (K.B.); ewa.brzezianska@umed.lodz.pl (E.B.-L.); 4 Institute of Medical Biology, Polish Academy of Sciences, 93-232 Lodz, Poland; jkryczka@cbm.pan.pl (J.M.K.); jboncela@cbm.pan.pl (J.B.); 5 Academic Laboratory of Movement and Human Physical Performance, Medical University of Lodz, 92-216 Lodz, Poland; info@gianlucapadula.pl; 6 Department of Orthopedics and Traumatology, University Clinical Hospital No. 2, Medical University of Lodz, 90-549 Lodz, Poland; krzysztof.nowak@umed.lodz.pl (K.N.); marcin.domzalski@umed.lodz.pl (M.D.)

**Keywords:** *ACAN*, *COL1A1*, *COL2A1*, osteoarthritis, real-time polymerase chain reaction, joint tissues, biomarkers

## Abstract

**Background:** Osteoarthritis (OA) patients experience poorer quality of life due to persistent symptoms and chronic pain. Unfortunately, the molecular basis of OA development is poorly understood, thus posing a major challenge for therapeutic interventions. The aim of this study was to compare the expression of genes encoding key cartilage proteins in OA-affected tissue from the relatively preserved lateral compartment of the knee joint in relation to knee OA advancement stage. This study focuses on molecular changes in individual areas of the knee, which may be important for the use of partial endoprostheses in patients. **Methods:** This study included 31 patients with primary knee OA. OA-affected tissues (medial compartment of the knee joint) and tissues from the relatively preserved lateral compartment were collected from each patient. Gene expression was assessed by real-time polymerase chain reaction (PCR). A protein–protein interaction network and functional visualization were prepared. **Results:** Compared with the relatively preserved lateral compartment, the more severely OA-affected medial compartment demonstrated significantly higher *COL1A1* mRNA expression in articular cartilage (*p* = 0.0045; Holm-adjusted *p* = 0.0269) and significantly higher *ACAN* mRNA expression in synovial tissue (*p* = 0.0091; Holm-adjusted *p* = 0.0458). In an exploratory subgroup analysis, patients with hypertension showed significantly higher *COL1A1* mRNA expression in cartilage from the more severely OA-affected medial compartment than in the relatively preserved lateral compartment (*p* = 0.00054). The verified proteins were found to be associated with proteins involved in the cartilage development. Based on The Gene Expression Omnibus (GEO) database, late-OA chondrocytes presented higher *ACAN* and *COL1A1*, and lower collagen type II (*COL2A1*) mRNA expression than early-OA ones. In the synovium, late-OA tissue from the relatively preserved lateral compartment showed lower *ACAN* and higher *COL1A1* mRNA expression than early-OA tissue. **Conclusions:** Numerous molecular changes are associated with OA. Cartilage and synovial membranes demonstrated similar changes in *ACAN* mRNA expression, whereas *COL1A1* and *COL2A1* displayed divergent expression patterns. The observed differences in gene expression depended on the part of tissue studied, biological features of patients and presence of comorbidities.

## 1. Introduction

While a number of joint diseases significantly worsen human quality of life, perhaps the most common is osteoarthritis (OA) [1,2,3]. Patients with OA are faced with chronic joint pain [4,5] resulting from inflammation in the synovial membrane [6] and deformations and abnormalities in the joint structure [7], such as joint space narrowing [8], cystic degeneration [9] and osteophyte formation [10]. These changes can limit or prevent joint function, resulting in reduced mobility [4], disability [1] and accelerated aging [11].

Osteoarthritis itself is known to affect the metabolism, structure, and functions of the entire joint tissue [12,13,14]. Most importantly, OA results in the degeneration of articular cartilage [13] and inflammation of the synovial membrane [15].

Many factors are believed to contribute to the development of OA; these include injuries causing inflammation [16], as well as aging [17] and obesity [1,18]. In addition, women are more prone to OA then men [3,17]. Although the molecular mechanism of OA development still remains unclear, Gene Ontology (GO) enrichment analysis highlights key biological processes, most notably collagen fibril organization and catabolism, pointing to a central role for collagen dysregulation in the disease pathogenesis [19]. Furthermore, ECM–receptor interaction pathways, pivotal for collagen formation, represent a key functional module among OA-associated genes, supporting bioinformatic evidence that altered collagen synthesis fuels OA onset and advancement [19].

The initial stages of OA are characterized by emerging inflammation [20] and increased production of proteolytic enzymes (metalloprotease [21,22] and aggrecanase [23,24]), leading to the degradation of aggrecan [23]; this results in the exposure of the collagen matrix to both lytic enzymes and mechanical stress. Collagen breakdown is also influenced by angiogenesis occurring during the development of OA [25] and the WNT signaling pathway in chondrocytes [26]. Crucial roles are also played by the degradation and suppressed synthesis of aggrecan and type II collagen, the two primary components of articular cartilage [24,27]. In physiological conditions, these two components cooperate to maintain the properties of cartilage; any imbalance leads to a reduction in load-bearing capacity, which is associated with OA [28].

Aggrecan, encoded by the *ACAN* gene, is a core protein, a chondroitin sulfate proteoglycan [29,30], which is responsible for ensuring the proper structure of cartilage [27] and ensure its resistance to compressive loads [27,31]. While the role of aggrecan in the articular cartilage has been extensively investigated, its function in the synovial membrane remains poorly understood. It has been proposed that aggrecan synthesized by the synovial mesenchymal progenitor cell (MPC) is secreted into the synovial fluid to maintain its viscosity and lubricating properties, potentially serving as a reservoir for neighboring joint structures [32].

During the development of OA, aggrecan is degraded by metalloprotease and aggrecanase. Metalloproteases are proteolytic enzymes that attack numerous cleavage sites within the G1-G2 domain of aggrecan, resulting in progressive aggrecan depletion, which is reportedly paralleled by decreased aggrecan gene expression [27].

Besides aggrecan, the most prominent structural component of articular cartilage is the fibrous protein collagen type II [25,27]. Encoded by the *COL2A1* gene, it is essential for maintaining cartilage structure and function [27,30,33]. In the synovial membrane, both the Col2-1 peptide and type II collagen fragments act as damage-associated molecular patterns (DAMPs) via the nuclear factor-kB (NF-kB) signaling, thereby triggering synovitis by stimulating the release of pro-inflammatory cytokine and metalloproteinase release [34].

Osteoarthritis development is also characterized by changes in the expression of collagen type I [35], encoded by *COL1A1*. Type I collagen is a stiff, fibrous protein that occurs in scar tissues and, unlike collagen type II, is rarely found in healthy chondrocytes. In the progression of OA, type I collagen is responsible for the development of fibrosis in the synovial membrane [36]. Its expression appears to increase significantly in the cartilage and synovium during the development of OA, and peaks in the final stage of OA [37,38].

Due to the diverse etiology of OA, its range of symptoms, and its substantial burden on daily patient functioning, there is a pressing need for molecular research to uncover new therapeutic and preventive targets. Future studies should particularly investigate the expression of genes encoding essential proteins in both cartilage and synovial tissue.

Following recent progress in understanding OA pathogenesis, the present study examines the effect of disease progression on *ACAN*, *COL2A1* and *COL1A1* mRNA expression in the cartilage and synovium, given their essential roles in preserving cartilage structure and function. We hypothesize that the expression of these genes is dynamically altered during OA development and progression. The aim of this study is to determine whether aggrecan and type II collagen mRNA levels decrease in the cartilage and synovium during OA progression, paralleled by an increase in type I collagen mRNA expression, reflecting cartilage degradation and tissue fibrosis.

Most studies linking *ACAN*, *COL2A1*, and *COL1A1* to osteoarthritis have been based on protein analysis, with fewer examining mRNA levels in OA patients. Furthermore, existing studies have mostly involved based on small patient groups and focused on either cartilage or the synovium separately. Although cartilage and synovium are functionally linked, no studies to date have compared gene expression levels in both tissues within in the same patient group, which forms the primary aim of the present study.

A key attribute of our work is its innovative approach of comparing OA-affected and tissues from the relatively preserved lateral compartment harvested from the same knee joint. Typically, degenerative changes within the knee joint do not progress uniformly: the medial compartment degenerates more frequently and rapidly than the lateral compartment. As a result, the cartilage in the medial compartment is generally thinner and exhibits more severe degenerative damage than in the lateral compartment [39,40]. Therefore, comparing the relatively preserved lateral compartment and the more severely OA-affected medial compartment from the same patient provides an internal control that reduces the influence of interindividual biological variability, which can otherwise obscure subtle molecular differences between joint compartments. The observed compartment-specific differences provide preliminary evidence of spatial heterogeneity in gene expression within the OA-affected knee. Although these findings may contribute to a better understanding of the molecular differences between relatively preserved and more severely affected joint compartments, the present exploratory study was not designed to assess their diagnostic, prognostic, or therapeutic utility. Further studies in larger, independent cohorts are required to determine whether these molecular differences have potential clinical relevance [41,42].

Our findings highlight key molecular alterations during osteoarthritis progression, suggesting that this targeted gene panel holds potential as a diagnostic biomarker. Given the complex interplay of immune, functional, and metabolic factors across cartilage, synovium, and subchondral bone, broader studies are necessary. Expanding this panel will be essential to establish robust transcriptional markers capable of tracking stage-specific OA pathology in both cartilage and synovial tissues. Although within-joint spatial comparisons remain relatively uncommon, they provide a valuable model for investigating the molecular heterogeneity of OA. Previous studies have demonstrated distinct gene-expression profiles between relatively preserved and more severely affected regions of the same knee. This approach may reduce interindividual biological variability while allowing the investigation of local molecular changes associated with different degrees of tissue degeneration. Nevertheless, standardized collection of spatially defined human joint tissues during arthroplasty is technically and logistically demanding, which may partly explain the limited number of such studies [39,40].

## 2. Materials and Methods

### 2.1. Studied Group

**Figure 1:**
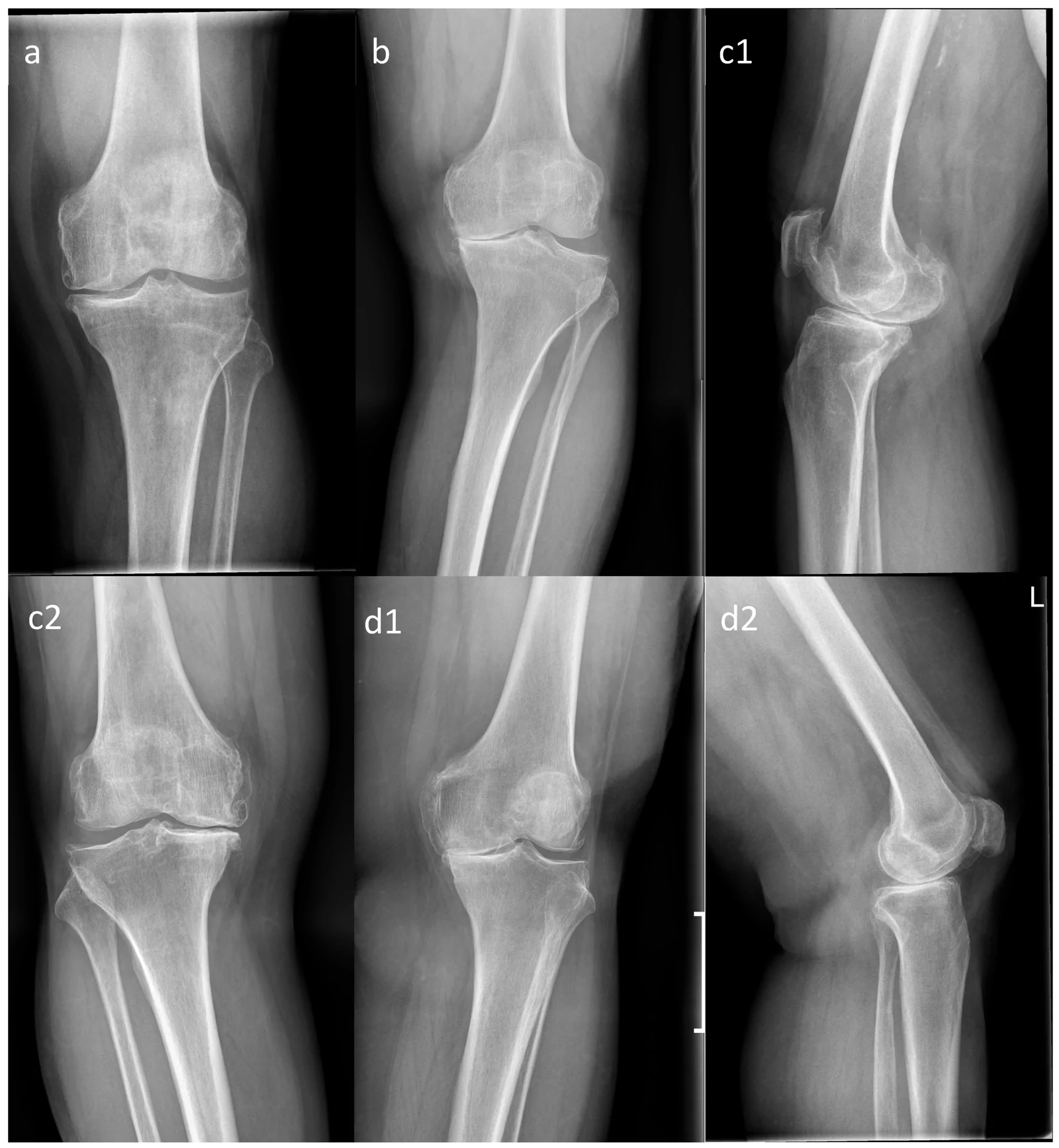
Examples of X-ray images of knee joints of patients participating in the study: patient no. 1, OA stage 4 (**a**), patient no. 2, OA stage 4 (**b**), patient no. 3, OA stage 4—1st projection (**c1**), 2nd projection (**c2**), patient no. 4, OA stage 4—1st projection (**d1**), 2nd projection (**d2**).

The study included 31 patients suffering from primary knee OA who were, referred for total knee arthroplasty. Qualification for surgery was confirmed through clinical assessment, medical history, physical examination and radiological evaluation according to the Kellgren-Lawrence scale. Examples of X-ray images showing both the lateral compartment (unaffected OA) and the medial compartment (OA-affected) of the knee joint are shown in Figure 1.

The inclusion criteria comprised primary knee OA with Kellgren–Lawrence grade 3 or 4 degenerative changes, chronic articular pain in the clinical picture, and possible reduction in knee joint mobility. Grade 3 is characterized by joint space narrowing, moderate osteophytes, and subchondral bone sclerosis whrereas grade 4 presents with severe joint space narrowing, prominent osteophytes, and marked sclerosis [43,44]. Tissue samples from patients at two different stages of OA severity were examined to evaluate changes in mRNA expression levels with disease progression rather than just disease onset The exclusion criteria comprised rheumatoid arthritis, psoriatic arthritis, hemophilia and neurological disorders. Written informed consent was obtained from each patient to participate in the study. Twenty-seven patients completed a survey regarding comorbidities, supplements use, and menopausal status whereas no survey data were obtained for four patients.

### 2.2. Biological Material

The study material consisted of cartilage tissue with the subchondral bone layer and synovial membrane, obtained intraoperatively during knee arthroplasty. Due to the non-uniform progression of osteoarthritic changes within the knee joint [39,40], biological material was obtained from both the medial and lateral compartments of the same knee. Since the medial compartment is known to undergo degenerative changes more frequently and rapidly, it was defined as OA-affected tissue. In contrast, as the lateral compartment typically exhibits less pronounced or no macroscopically evident degenerative changes, was used as an internal comparator and was operationally defined as “unaffected OA tissue” throughout the manuscript. Importantly, the term “unaffected” refers to the relatively preserved macroscopic appearance of the lateral compartment and does not imply histologically healthy or OA-free tissue. Each biological sample (1 cm^3^ fragments in the case of cartilage tissues) was collected by an orthopedic traumatologist performing total knee arthroplasty. The procedure was carried out at the Department of Orthopedics and Traumatology, University Clinical Hospital No. 2 of the Military Medical Academy—Central Veterans Hospital in Lodz.

### 2.3. Research Methods

#### 2.3.1. Molecular Analysis

The molecular analysis was performed at the Department of Biomedicine and Genetics, Medical University of Lodz.

Total RNA was isolated from the collected tissues specimens. The sample were then subjected to reverse transcription (RT), and mRNA expression levels of *ACAN*, *COL1A1* and *COL2A1* were assessed using real-time polymerase chain reaction (PCR).

#### 2.3.2. RNA Isolation

The pure total RNA fraction was isolated from cartilage and synovial membrane tissues using the column-based mirVana Isolation Kit (Life Technologies, Carlsbad, CA, USA) according to the manufacturer’s recommendations.

#### 2.3.3. Quantitative and Qualitative Assessment of Isolated Total RNA

The total RNA concentrations of each sample was determined in triplicate using a BioPhotometerTM Plus spectrophotometer (Eppendorf, Hamburg, Germany).

#### 2.3.4. RT Reaction

Reverse transcription (RT) was performed using a High-Capacity cDNA Reverse Transcription Kit (Applied Foster City, CA, USA) in a volume of 20 µL, according to the manufacturer’s instructions. The reaction mixture contained total RNA, 10-fold concentrated TaqMan RT Buffer, ribonuclease inhibitor (RNase inhibitor—RI), a mixture of deoxyribonucleotide triphosphates (dNTP), oligonucleotide primers (random hexamer primers), and nuclease-free water. The reaction was carried out in a Personal Thermocycler (Eppendorf, Hamburg, Germany) with the following thermal cycle: 20 °C for 10 min, 37 °C for 2 h, and 85 °C for 5 s. Subsequently, samples were cooled to 4 °C, and the obtained complementary DNA (cDNA) was stored at −20 °C.

#### 2.3.5. Real-Time PCR

The relative expression (RQ) of the target genes was evaluated using real-time PCR with the 7900HT Fast Real-Time PCR System (Applied Biosystems, Carlsbad, CA, USA). RQ values were determined in triplicate using the following TaqMan probes (Applied Biosystems, Carlsbad, CA, USA): Hs00153936_m1 for *ACAN* mRNA, Hs00164004_m1 for *COL1A1* mRNA and Hs00264051_m1 for *COL2A1* mRNA. The hypoxanthine-guanine phosphoribosyltransferase (HPRT1) gene, previously confirmed as a stable reference gene in both cartilage [45] and synovium [46], was used as the endogenous control TaqMan Hs02800695_m1 probe).

During real-time amplification, the samples were incubated at 50 °C for 2 min, and at 94.5 °C for 10 min. Subsequently, 40 cycles were performed with the following thermal sequence: 97 °C for 30 s, and 59.7 °C for 1 min. Standard total RNA from human chondrocytes (ScienCell Research Laboratories, USA) was used as a calibrator.

The RQ of the tested genes was compared with the endogenous control using the ΔΔC_T_ (C_T_—threshold cycle) method. The following formula was used: ΔΔC_T_ = ΔC_T_ of the test sample—ΔC_T_ of the calibrator. It was assumed that an RQ > 1 indicated an increase in gene expression, while an RQ < 1 represented a decrease in expression relative to the calibrator.

The primary endpoint was to determine changes in the expression of *ACAN*, *COL1A1* and *COL2A1* mRNA in knee cartilage and synovial tissues from patients with OA. The study aimed was to assess the impact of disease progression on gene expression to identify potential molecular markers for OA diagnosis. The primary endpoint was the difference in *ACAN*, *COL1A1* and *COL2A1* mRNA expression between tissue obtained from the more severely OA-affected medial compartment and the relatively preserved lateral compartment. Analyses according to OA stage and patient characteristics, including sex, diabetes and hypertension, as well as correlation analyses, were considered exploratory and hypothesis-generating.

### 2.4. Protein–Protein Interaction Network

A Protein–Protein Interaction (PPI) network was created and visualized using the STRING version 12.0 online platform (https://string-db.org/, accessed on 24 April 2024), as previously described [47,48].

The Gene Ontology (GO) database was chosen for PPI analysis over the Kyoto Encyclopedia of Genes and Genomes (KEGG). The GO database provides a hierarchical and comprehensive functional annotation framework that enables enrichment analysis and interpretation of network modules of biological specificity at multiple levels [49]. Also, as GO annotations underpin functional enrichment in visualization tools such as STRING, it is particularly well-suited for network-based studies [50,51]. Moreover, GO accommodates protein multifunctionality and overlapping biological roles, whereas KEGG pathways tend to impose linear biological structures that may oversimplify analyzed network hubs. Furthermore, KEGG is pathway-centric and optimized for reconstructing canonical metabolic or signaling pathways rather than for abstract interaction network analysis, which may limit annotation coverage for heterogeneous PPI datasets [52].

### 2.5. Functional Enrichment Analysis

Functional enrichment analysis of Biological Process (BP) and Molecular Functions (MF) was performed and visualized using the FunRich software (v3.1.3) (http://www.funrich.org/, accessed on 24 April 2024) supported by the GO database (http://geneontology.org/, accessed on 24 April 2024), analogous to our previous work [48].

### 2.6. Database Accession

Gene expression profiles with accession numbers GSE179716 (https://www.ncbi.nlm.nih.gov/geo/query/acc.cgi?acc=GSE179716, accessed on 24 April 2024), and GSE206848 (https://www.ncbi.nlm.nih.gov/geo/query/acc.cgi?acc=GSE206848, accessed on 24 April 2024) were downloaded from The Gene Expression Omnibus (GEO) database (http://www.ncbi.nlm.nih.gov/geo/) (accessed on 24 April 2024) and analyzed as described previously [47,48]. All data were processed using the GEO2R online analytical tool (# Version info: R 4.2.2, Biobase 2.58.0, GEOquery 2.66.0, limma 3.54.0) [47,48]. The mRNA expression of selected genes was calculated and visualized using Orange Data Mining software v. 3.31.1 (https://orangedatamining.com/, accessed on 24 April 2024). Spearman’s correlation matrix of chosen genes was calculated and visualized using the JASP software v. 0.16.0.0 (https://jasp-stats.org/, accessed on 24 April 2024) based on data from GSE179716 and GSE206848 [47].

### 2.7. Ethics of Scientific Research

Official consent for research on human subjects was obtained from the Bioethics Committee of the Medical University of Lodz, number RNN/199/19/KE. In addition, all study participants voluntarily gave their informed written consent to take part.

### 2.8. Statistical Analyses

Data were expressed as the median, lower and upper quartiles. The normality of the data distribution was assessed using the Shapiro–Wilk test. Comparisons of gene expression between the more severely OA-affected medial compartment and the relatively preserved lateral compartment obtained from the same patient were treated as paired comparisons. For non-normally distributed paired data, the Wilcoxon signed-rank test was used, whereas normally distributed paired data were analyzed using the paired-samples *t*-test. Because the primary endpoint comprised six paired comparisons (three genes analyzed separately in articular cartilage and synovial tissue), multiplicity within the primary analysis was addressed using the Holm procedure. Both nominal and multiplicity-adjusted *p*-values were considered, with an adjusted *p*-value < 0.05 regarded as statistically significant. Exploratory subgroup and correlation analyses were not adjusted for multiple testing and were interpreted as hypothesis-generating. The nonparametric Kruskal–Wallis test was used to compare gene expression between multiple independent samples that were not normally distributed. Differences between specific variables were evaluated by multiple comparisons of mean ranks.

The degree of correlation was calculated using Spearman’s test, for non-normally distributed individual variables, or Pearson’s test, for normally distributed variables. A *p*-value of < 0.05 was considered statistically significant. A power analysis was also performed. Statistical analyses were conducted with the STATISTICA software (version 13.1, StatSoft).

## 3. Results

### 3.1. Biometrical Features of the Studied Group

The study group consisted of 31 patients with primary OA: 21 women and 10 men. The mean age of the patients was 66 ± 7 years. Their clinical characteristics are presented in Table 1.

### 3.2. Molecular Analysis

#### 3.2.1. Differences in the Relative Expression (RQ) of Selected Genes in Articular Cartilage

The RQ values of *ACAN*, *COL1A1*, and *COL2A1* mRNA in the unaffected and cartilage from the more severely OA-affected medial compartment tissue are presented in Table 2. The analysis was performed using the Wilcoxon test.

The data indicate that *ACAN* mRNA expression was upregulated (RQ > 1) in 100% of Cartilage from the more severely OA-affected medial compartment samples, and *COL2A1* mRNA was upregulated in OA-affected tissue 96.77%. Comparable percentages of upregulation (RQ > 1) were likewise detected in the corresponding tissue from the relatively preserved lateral compartment samples No statistically significant differences in *ACAN* or *COL2A1* mRNA expression were observed between cartilage from the more severely OA-affected medial compartment and the relatively preserved lateral compartment (*p* > 0.05) (Table 2). Regarding disease progression, no statistically significant differences in *ACAN* or *COL2A1* mRNA expression were observed between stage 3 or stage 4 Cartilage from the more severely OA-affected medial compartment and the relatively preserved lateral compartment (*p* > 0.05). Furthermore, *ACAN* and *COL2A1* mRNA expression did not differ significantly between stage 3 and stage 4 Cartilage from the more severely OA-affected medial compartment (*p* > 0.05) (Table 2).

*COL1A1* mRNA expression was upregulated (RQ > 1) in 80.65% of the Cartilage from the more severely OA-affected medial compartment samples and in 51.61% of cartilage from the relatively preserved lateral compartment controls. Significantly higher *COL1A1* mRNA expression was found in the Cartilage from the more severely OA-affected medial compartment compared to the cartilage from the relatively preserved lateral compartment (median RQ: 5.04 and 1.06, respectively) (*p* = 0.0045; Holm-adjusted *p* = 0.0269, power = 0.829) (Table 2).

Regarding disease progression, no statistically significant difference in *COL1A1* mRNA expression was observed between stage 3 Cartilage from the more severely OA-affected medial compartment and the relatively preserved lateral compartment (*p* > 0.05). In contrast significantly higher expression was noted in OA-affected (stage 4) compared to cartilage from the relatively preserved lateral compartment (median RQ: 5.27 and 1.06, respectively; *p* = 0.0075, power = 0.59).

No statistically significant difference in *COL1A1* mRNA expression was observed between stage 3 and stage 4 Cartilage from the more severely OA-affected medial compartment (*p* > 0.05) (Table 2).

#### 3.2.2. Correlation Between Selected Gene Expression and OA Severity Compared to Cartilage from the Relatively Preserved Lateral Compartment

The correlation of *ACAN*, *COL1A1* and *COL2A1* mRNA expression (RQ) with OA stages are presented in Table 3. The analysis was performed using Spearman’s Test.

A positive correlation was observed between *ACAN* mRNA expression in the Cartilage from the more severely OA-affected medial compartment (stage 3 + 4) and cartilage from the relatively preserved lateral compartment (rho = 0.396; *p* = 0.027, power = 0.6156). Additionally, a significant, strong positive correlation was noted between *ACAN* mRNA expression in OA-affected (stage 4) cartilage and cartilage from the relatively preserved lateral compartment (rho = 0.517; *p* = 0.034, power = 0.5987) (Table 3).

A very weak positive correlation for *COL1A1* mRNA expression was found between all OA-affected medial compartment (stage 3 + 4) and cartilage from the relatively preserved lateral compartment (*p* > 0.05). An intermediate positive correlation was also noted for *COL1A1* mRNA expression between the OA-affected (stage 3) tissue and cartilage from the relatively preserved lateral compartment (*p* > 0.05) whereas a weak negative correlation was also found between OA-affected medial compartment (stage 4) tissue and unaffected with regard to *COL1A1* mRNA expression. None of these correlations were statistically significant (*p* > 0.05) (Table 3).

Weak positive correlations were noted for *COL2A1* mRNA expression between the Cartilage from the more severely OA-affected medial compartment (stage 3 + 4) and cartilage from the relatively preserved lateral compartment, and between the OA-affected medial compartment (stage 4) cartilage and cartilage from the relatively preserved lateral compartment. A very weak positive correlation was found between OA-affected medial compartment (stage 3) cartilage and cartilage from the relatively preserved lateral compartment. However, none of these correlations achieved statistical significance (*p* > 0.05). (Table 3).

#### 3.2.3. The Effect of on Sex on Differences in RQ, and the Correlation Between the Expression of Selected Genes in OA-Affected Medial Compartment and Relatively Preserved Lateral Compartment Articular Cartilage

The relative expression (RQ) of *ACAN*, *COL1A1*, and *COL2A1* mRNA in relatively preserved lateral compartment and cartilage from the more severely OA-affected medial compartment with regard to sex are presented in Table 4. The analysis was performed using Wilcoxon Test.

In the female group, no statistically significant differences in *ACAN*, *COL1A1*, or *COL2A1* mRNA expression were observed between cartilage obtained from the more severely OA-affected medial compartment and the relatively preserved lateral compartment (*p* > 0.05; Wilcoxon signed-rank test) (Table 4).

In the male group, no statistically significant differences in *ACAN* or *COL2A1* mRNA expression were observed between cartilage obtained from the more severely OA-affected medial compartment and the relatively preserved lateral compartment (*p* > 0.05; Wilcoxon signed-rank test). In contrast, *COL1A1* mRNA expression was significantly higher in cartilage from the more severely OA-affected medial compartment than in cartilage from the relatively preserved lateral compartment (median RQ: 5.85 vs. 0.94, respectively; *p* = 0.0366; power = 0.55; Wilcoxon signed-rank test) (Table 4).

Male patients exhibited higher *ACAN* and *COL2A1* mRNA expression, as well as marginally higher *COL1A1* mRNA levels, in Cartilage from the more severely OA-affected medial compartment compared to females (Table 4).

The correlation of *ACAN*, *COL1A1*, and *COL2A1* mRNA expression between the OA-affected medial compartment and cartilage from the relatively preserved lateral compartment according to sex is presented in Table 5. The analysis was performed using both Spearman’s and Pearson’s test.

In the female group, no statistically significant correlations were observed between gene expression in cartilage from the relatively preserved lateral compartment and the more severely OA-affected medial compartment for *ACAN*, *COL1A1*, or *COL2A1* (*p* > 0.05) (Table 5).

Similarly, in the male group, no statistically significant correlations were observed for *ACAN*, *COL1A1*, or *COL2A1* mRNA expression between the relatively preserved lateral compartment and the more severely OA-affected medial compartment (*p* > 0.05) (Table 5).

#### 3.2.4. The Influence of Metabolic Comorbidities on the Relative Expression (RQ) of Selected Genes in OA-Affected Medial Compartment and Unaffected Articular Cartilage

The relationship between relative expression (RQ) of *ACAN*, *COL1A1*, and *COL2A1* mRNA in OA-affected medial compartment and cartilage from the relatively preserved lateral compartment and the presence of hypertension and diabetes is presented in Table 6. Four patients (*n* = 4) lacking data on metabolic comorbidities were excluded from the analysis. The statistical analysis was performed using the Kruskal–Wallis Test.

No statistically significant differences in *ACAN*, *COL1A1*, or *COL2A1* mRNA expression were observed between patients with and without diabetes, either in cartilage from the relatively preserved lateral compartment or in cartilage from the more severely OA-affected medial compartment (*p* > 0.05) (Table 6).

Similarly, no statistically significant differences in *ACAN*, *COL1A1*, or *COL2A1* mRNA expression were observed between patients with and without hypertension within either compartment (*p* > 0.05) (Table 6).

In patients with hypertension, *COL1A1* mRNA expression was significantly higher in cartilage from the more severely OA-affected medial compartment than in cartilage from the relatively preserved lateral compartment tissues (*p* = 0.00054).

#### 3.2.5. Analysis of Differences in the Expression Level (RQ) of Selected Genes in the Synovium

The relative expression (RQ) of *ACAN*, *COL1A1*, and *COL2A1* mRNA in the OA-affected and synovial tissue from the relatively preserved lateral compartment are presented in Table 7. The analysis was performed using the Wilcoxon signed-rank test for paired samples.

The expression of *ACAN* mRNA and *COL1A1* mRNA was downregulated (RQ < 1) in 93.55% and 96.77% of OA synovial membranes respectively and in all (100%) unaffected membranes. *COL2A1* mRNA expression was upregulated (RQ > 1) in 64.52% of the OA-affected synovial membranes and in 74.19% of synovial tissue from the relatively preserved lateral compartment.

*ACAN* mRNA expression was significantly higher in synovial tissue obtained from the more severely OA-affected medial compartment than in tissue from the relatively preserved lateral compartment (*p* = 0.0091; Holm-adjusted *p* = 0.0458, power = 0.755). No statistically significant differences in *COL1A1* or *COL2A1* mRNA expression were observed between synovial tissue from the more severely OA-affected medial compartment and the relatively preserved lateral compartment (*p* > 0.05) (Table 7).

Regarding disease progression, we observed no statistically significant differences in *ACAN*, *COL1A1*, or *COL2A1* mRNA expression when stage 3 and stage 4 OA-affected synovial tissue were compared separately with the relatively preserved lateral compartment (*p* > 0.05). Moreover, no statistically significant differences in the expression of these genes were observed between stage 3 and stage 4 OA-affected synovial tissue (*p* > 0.05) (Table 7).

#### 3.2.6. Correlation Between the Expression of the Selected Genes and the Severity of OA Compared to Synovial Tissue from the Relatively Preserved Lateral Compartment

The correlation between *ACAN*, *COL1A1*, and *COL2A1* mRNA expression (RQ) and OA stages are presented in Table 8. The statistical analysis was performed using Spearman’s test.

For *ACAN* mRNA expression, a significant moderate positive correlation was observed between the relatively preserved lateral compartment and the OA-affected medial compartment for the combined stage 3 + 4 group (rho = 0.464, *p* = 0.009; power = 0.7713). A significant strong positive correlation was also observed in the stage 3 subgroup (rho = 0.696, *p* = 0.003; power = 0.8933), whereas the correlation in the stage 4 subgroup was not statistically significant (*p* > 0.05) (Table 8).

For *COL1A1* mRNA expression, weak correlations were observed between the relatively preserved lateral compartment and the OA-affected medial compartment; however, none of these correlations reached statistical significance (*p* > 0.05) (Table 8).

For the *COL2A1* mRNA, significant, strong positive correlations were observed between OA-affected and synovial tissue from the relatively preserved lateral compartment (rho = 0.634, *p* < 0.001, power = 0.9805), as well as between OA-affected (stage 3) and synovial tissue from the relatively preserved lateral compartment (rho = 0.675, *p* = 0.008, power = 0.8070), and between OA-affected (stage 4) and synovial tissue from the relatively preserved lateral compartment (rho = 0.551, *p* = 0.022, power = 0.6677) (Table 8).

#### 3.2.7. Sex-Dependent Variations in Relative Expression (RQ) and Gene Correlations in OA-Affected vs. Synovial Tissue from the Relatively Preserved Lateral Compartment

The relative expression (RQ) of *ACAN*, *COL1A1*, and *COL2A1* mRNA in synovial tissue obtained from the relatively preserved lateral compartment and the more severely OA-affected medial compartment, stratified by sex, is presented in Table 9. The statistical analysis was performed using the Wilcoxon signed-rank test for paired samples.

In the female group, *ACAN* mRNA expression was significantly higher in synovial tissue from the more severely OA-affected medial compartment than in the relatively preserved lateral compartment (median RQ: 0.023 and 0.003, respectively; *p* = 0.0106; power = 0.74; Wilcoxon signed-rank test). In the male group, *ACAN* mRNA expression did not differ significantly between the medial and lateral compartments (*p* > 0.05; Wilcoxon signed-rank test) (Table 9).

Regarding *COL1A1* mRNA expression, no statistically significant differences were observed between the more severely OA-affected medial compartment and the relatively preserved lateral compartment in either women or men (*p* > 0.05; Wilcoxon signed-rank test) (Table 9).

Similarly, we found no statistically significant differences in *COL2A1* mRNA expression between the medial and lateral compartments in either women or men (*p* > 0.05; Wilcoxon signed-rank test) (Table 9).

The correlation of *ACAN*, *COL1A1*, and *COL2A1* mRNA expression between OA-affected and synovial tissue from the relatively preserved lateral compartment with regard to sex are presented in Table 10. The statistical analysis was performed using Spearman’s test.

In the female group, a significant strong positive correlation was found between OA-affected (stage 3 + 4) and synovial tissue from the relatively preserved lateral compartment for the *ACAN* mRNA expression (rho = 0.600, *p* = 0.005, power = 0.8544), anon-significant weak negative correlation for *COL1A1* mRNA (*p* > 0.05), and a significant moderate positive correlation for the *COL2A1* mRNA (rho = 0.400, *p* = 0.041, power = 0.4537) (Table 10).

On the other hand, in the male group, anon-significant weak positive correlation was noted between OA-affected (stage 3 + 4) and synovial tissue from the relatively preserved lateral compartment for *ACAN* mRNA expression (*p* > 0.05), a statistically nonsignificant weak negative correlation for *COL1A1* mRNA (*p* > 0.05), but a significant very strong positive correlation for *COL2A1* mRNA (rho = 0.900, *p* < 0.001, power = 0.9846) (Table 10).

#### 3.2.8. Influence of Metabolic Comorbidities on the Relative Expression (RQ) of Selected Genes in OA-Affected and Unaffected Articular Synovium

The influence of metabolic comorbidities on the relative expression (RQ) of *ACAN*, *COL1A1*, and *COL2A1* mRNA in unaffected and synovial tissue from the more severely OA-affected medial compartment was assessed in patients with available clinical data. Four patients with missing information on metabolic comorbidities were excluded from this analysis. No statistically significant differences in the expression of the investigated genes were observed according to diabetes or hypertension status (all *p* > 0.05).

### 3.3. Analysis of Protein–Protein Interactions in Cartilage and Synovium Proteins

**Figure 2:**
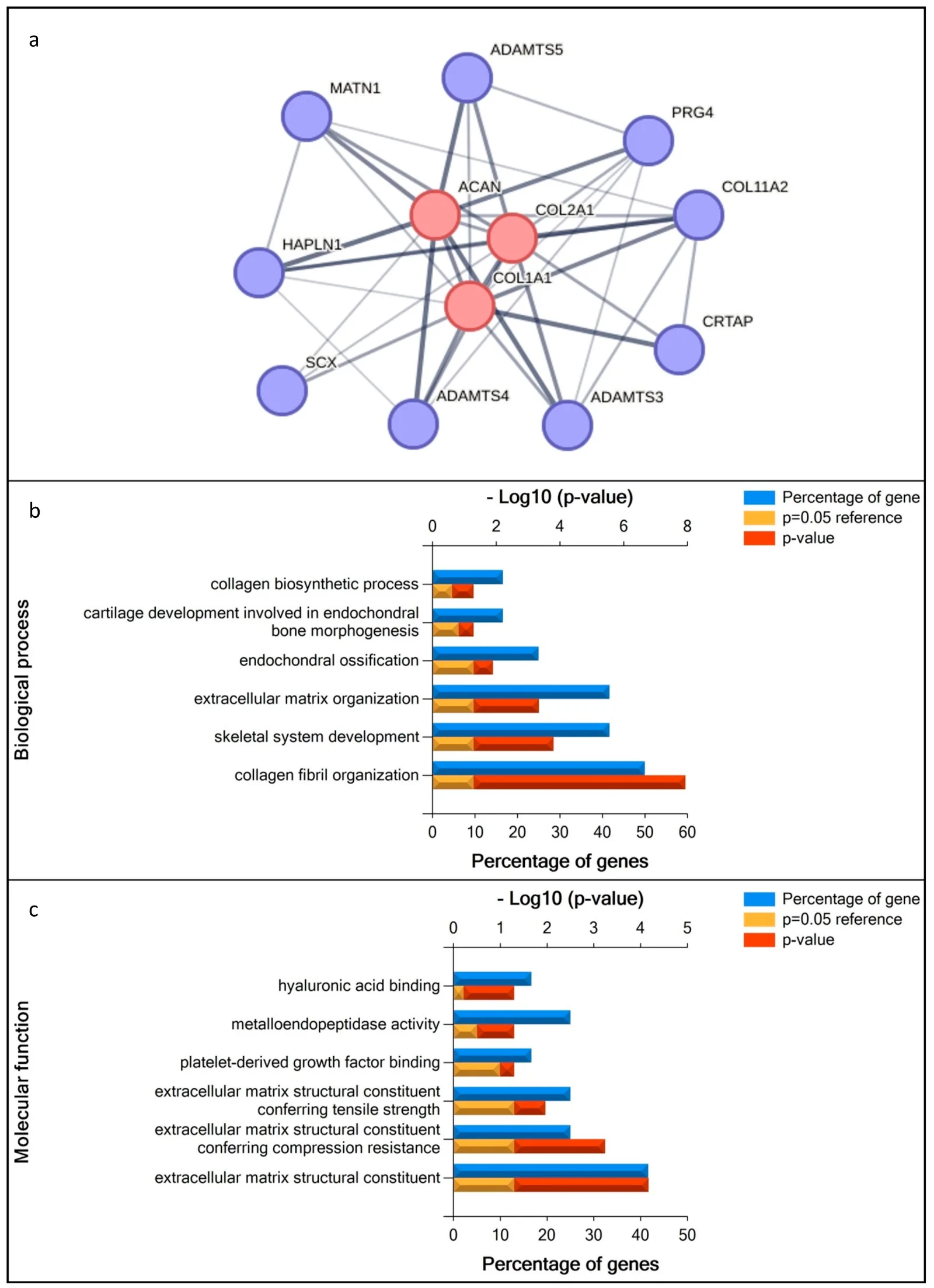
PPI network of COL1A1, COL2A1, and ACAN enhanced by known associated proteins. The PPI network is shown in red, and enhancements by known co-occurring proteins are shown in blue. The PPI network was created and visualized by the STRING version 12.0 online platform (**a**). Functional enrichment analysis of PPI network proteins in Biological Processes (**b**) and Molecular Functions (**c**) was performed using FunRich software (v3.1.3) supported by the GO database. *ADAMTS3*—A Disintegrin and Metalloproteinases with Thrombospondin motifs type 3; *ADAMTS4*—A Disintegrin and Metalloproteinases with Thrombospondin motifs type 4; *ADAMTS5*—A Disintegrin and Metalloproteinases with Thrombospondin motifs type 5; *COL11A2*—collagen type XI; *CRTAP*—cartilage-related protein; *HAPLN1*—hyaluronan-proteoglycan-linking protein 1; *MATN1*—matrilin-1; *PRG4*—proteoglycan 4; *SCX*—transcription factor scleraxis.

The molecular connotation and mutual interaction of *COL1A1*, *COL2A1*, and *ACAN* were determined using a PPI network based on the STRING (version 12.0) online platform (Figure 2a). The primarily created network (shown in red) was further enhanced by known proteins (shown in blue) that are closely related to the *COL1A1*, *COL2A1*, and *ACAN*, thus filling the gaps in the network, as described by Makondi et al. [53]. The created network was subjected to functional enrichment analysis (Biological Process and Molecular Functions), and visualized using FunRich software (v3.1.3) and the GO database (Figure 2b,c). The results confirmed that the proteins of interest play significant roles in cartilage development and skeletal system organization, via collagen biosynthesis, hyaluronic acid deposition, regulation of metalloprotein activity, and extracellular matrix remodeling.

Having established the PPI network of *COL1A1*, *COL2A1*, and *ACAN* mRNA expression, the genes coding the top networking proteins were analyzed using the OA-affected tissue data deposited in the GEO database. Two datasets were chosen for this analysis. The GSE179716 dataset consists of transcriptomic data from chondrocytes isolated from three patients with early-stage of OA and three with late-stage of OA; obtained by University of Pittsburgh (Pittsburgh, PA, USA) using the Illumina NextSeq 500 platform. Preserved and damaged cartilage samples were identified based on the Outerbridge score (macro appearance) and Osteoarthritis Research Society International (OARSI) score (histological), and isolated from patients who underwent total knee joint arthroplasty (https://www.ncbi.nlm.nih.gov/geo/query/acc.cgi?acc=GSE179716, accessed on 29 June 2026).

The GSE206848 dataset includes transcriptomic data from the synovium isolated from seven patients with normal, non-synovial tissue from the more severely OA-affected medial compartment, and seven patients with synovial tissue from the more severely OA-affected medial compartment obtained during knee replacement surgery; the data were obtained by the Grossman School of Medicine (New York, NY, USA) using the Affymetrix Human Genome U133 Plus 2.0 platform (https://www.ncbi.nlm.nih.gov/geo/query/acc.cgi?acc=GSE206848, accessed on 29 June 2026).

**Figure 3:**
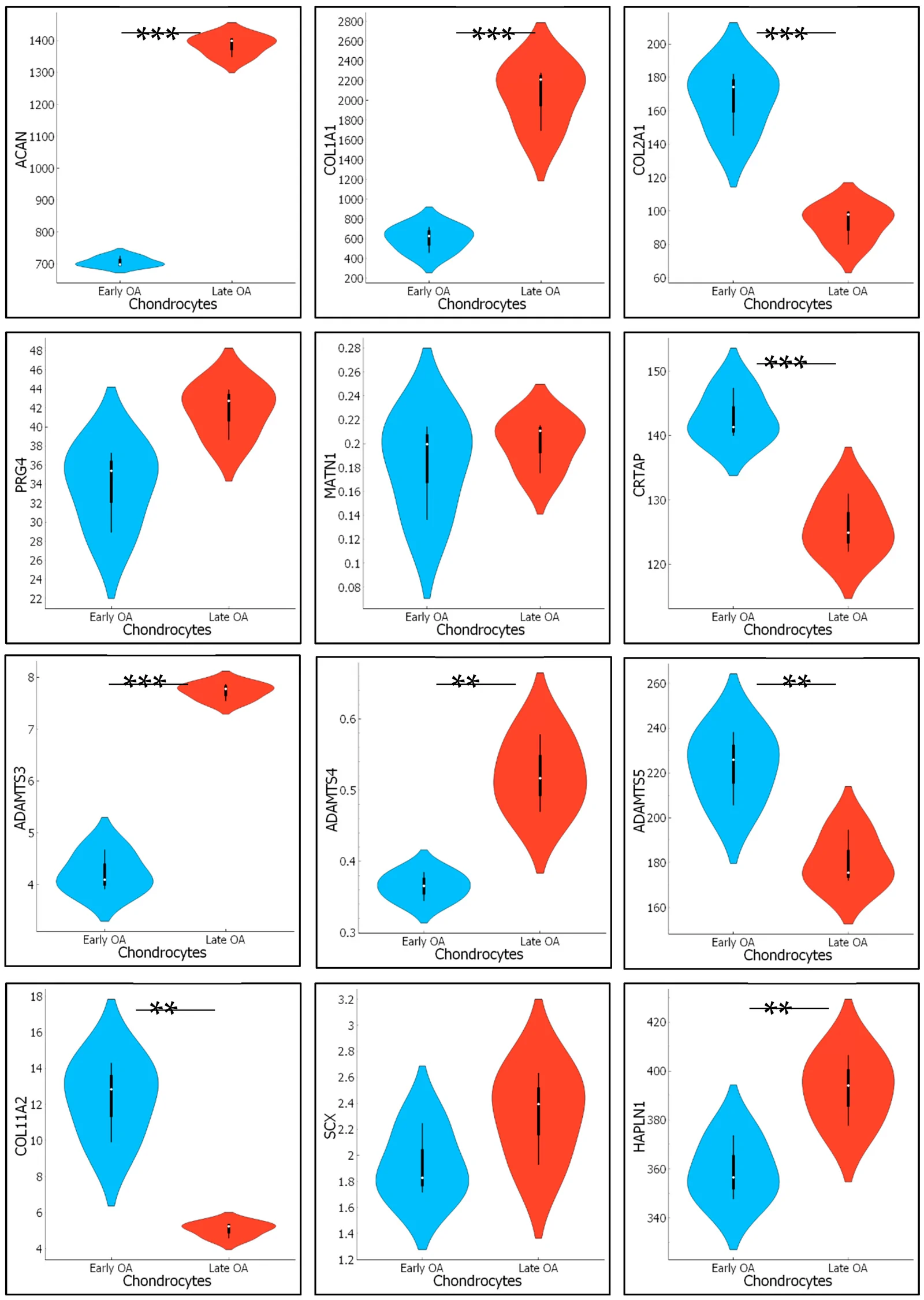
mRNA expression of chosen genes in chondrocytes isolated from late and early OA-affected knee joints. Transcriptomic data were downloaded from the GSE179716 dataset of the GEO database. Analysis and visualization were performed using the Orange Data Mining platform. The data was subjected to the Shapiro–Wilk test of normality, followed by the Mann–Whitney test (for non-normally distributed data) or the *t*-test (for normally distributed data); ** *p* < 0.005, and *** *p* < 0.001.

**Figure 4:**
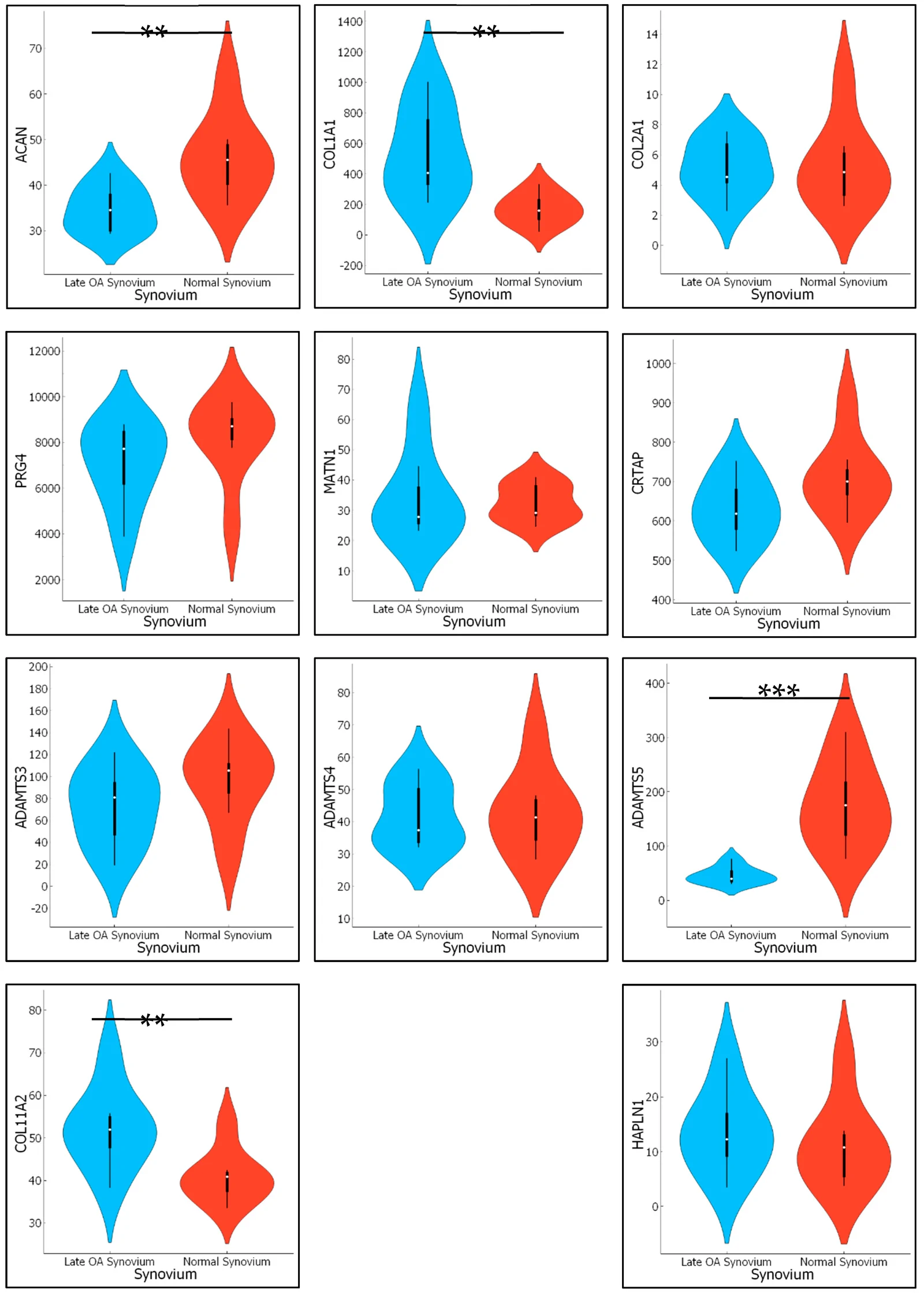
mRNA expression of chosen genes in synovium isolated from normal and OA-affected knee joints. Transcriptomic data were downloaded from the GSE206848 dataset of the GEO database. Analysis and visualization were performed using the Orange Data Mining platform. The data was subjected to the Shapiro–Wilk test of normality, followed by the Mann–Whitney (for non-normally distributed data) or the *t*-test (for normally distributed data); ** *p* < 0.005, and *** *p* < 0.001.

The Orange Data Mining platform was used to calculate and visualize the mRNA expression in the chosen genes in chondrocytes (Figure 3) and synovium (Figure 4).

Additionally, a Spearman’s correlation matrix was developed using the JASP 0.16.0.0 software (Figure S1 and Figure S2). In OA-affected chondrocytes, *ACAN*, *COL1A1*, *ADAMTS3*, *ADAMTS4*, and *HAPLN1* mRNA expression was significantly increased in late-stage OA compared to early-stage OA, whereas *COL2A1*, *ADAMTS5*, *CRTAP*, and *COL11A2* expression was significantly decreased. Additionally, *COL1A1* expression demonstrated a strong positive correlation with *ACAN*, *PRG4*, *SCX* and *ADAMTS4*, and a negative correlation with *CRTAP* and *ADAMTS5*. Interestingly, in the synovium, only four of the chosen genes presented significant changes, with *COL1A1* and *COL11A1* being up-regulated in Synovial tissue from the more severely OA-affected medial compartment, and *ACAN* and *ADAMTS5* being down-regulated. *COL1A1* mRNA expression was negatively correlated with *ACAN*, *CRTAP* and *ADAMTS5*.

## 4. Discussion

Our findings confirm that significant alterations in *ACAN*, *COL2A1*, and *COL1A1* expression occur during OA progression.

The present study takes a novel approach by comparing gene expression in OA-affected tissue collected from the medial compartment of the knee joint, with tissue from the relatively preserved lateral compartment from the lateral compartment of the same knee. In contrast most studies to date have compared gene expression between a group of patients with OA and an independent group without OA [54,55,56].

This approach allows for effective control of interindividual variability and enables a direct focus exclusively on local, differences related to the disease.

This observational study identified compartment- and tissue-specific differences in the expression of selected extracellular-matrix-related genes in advanced knee OA. In particular, *COL1A1* expression differed between the more severely affected medial cartilage and the relatively preserved lateral compartment, while *ACAN*, *COL1A1* and *COL2A1* showed heterogeneous expression patterns depending on tissue type and disease characteristics. These observations are consistent with spatial molecular heterogeneity within the osteoarthritic knee. However, because of the limited sample size, exploratory subgroup analyses, multiple exploratory comparisons, and the candidate-gene design, these results should be regarded as preliminary and hypothesis-generating. Larger, adequately powered studies incorporating broader molecular profiling and independent validation cohorts are required to determine the biological and potential clinical relevance of these observations [57,58,59].

Against this background, the present study aimed to characterize compartment-specific expression patterns of *ACAN*, *COL1A1*, and *COL2A1* in cartilage and synovium from OA-affected knees. A key finding was that *COL1A1* mRNA expression is significantly higher in Cartilage from the more severely OA-affected medial compartment compared to cartilage from the relatively preserved lateral compartment, and that *ACAN* mRNA was significantly higher in synovial tissue from the more severely OA-affected medial compartment compared to synovial tissue from the relatively preserved lateral compartment. Furthermore, in patients with hypertension, Cartilage from the more severely OA-affected medial compartment exhibited significantly higher *COL1A1* mRNA expression than cartilage from the relatively preserved lateral compartment, whereas, this difference did not reach statistical significance in non-hypertensive patients.

Although previous studies have examined *ACAN*, *COL2A1*, and *COL1A1* expression in cartilage and synovium, their results remain inconsistent and strongly dependent on experimental conditions [38,54,55,60,61,62]. As age, sex, and BMI are known to be key risk factors in the development of OA [63,64], these variables were also considered in our study design.

Epidemiological data establish that osteoarthritis is more common in women than in men [2,3,17] and in older individuals [1,3], with its incidence peaking at ages 50–59 [2] or 70–79 years depending on the study [17]. This pattern suggests that the majority of women suffering from OA are postmenopausal, which may also account for the observed sex differences in disease presentation. These findings were carefully considered in the present study. Bay-Jensen et al. [65] reported higher levels of C-terminal telopeptide of type II collagen (CTX-II), a marker of type II knee degradation, in the urine of postmenopausal women compared to premenopausal controls, suggesting that cartilage degradation may be enhanced by lower estrogen levels. Similarly, Jin et al. [66] noted that low serum levels of endogenous sex hormones (estradiol, progesterone, and testosterone) in women were associated with synovitis and knee joint effusion.

In addition, a number of studies have found that specific structural characteristics of the knee joint differ between women and men, such as knee joint dimensions, medial-to-lateral ratios [67], the ratio of medial-lateral to anteroposterior dimensions [68], as well as that women tend to demonstrate lower congruence index values in the medial tibiofemoral joint than men [69]. These anatomical variations may influence the development of OA. A 1998 study of joint biomechanics found women’s joints to be exposed to greater mechanical work during gait, which could account for the faster wear of articular cartilage and the subsequent development of OA [70].

The mean BMI of our study cohort was 33.03 kg/m^2^, corresponding to class I obesity. Elevated body weight is well known to influence the development of various OA-related symptoms, such as joint cartilage degradation [71,72] and horizontal atrophy at the cartilage-bone junction [73]. Furthermore, obese patients are more likely to experience severe [18] and bilateral knee pain [5]. Obesity has also been associated with chronic microinflammation [18], high levels of serum cartilage oligomeric matrix protein (sCOMP), a marker of OA [74], and increased infrapatellar fat pads (IFP), which may result in higher levels of pro-inflammatory adipokines [75]. Also, while physical activity is recommended to relieve OA symptoms [76], OA patients with obesity have been found to engage in less physical activity than non-obese individuals [5,18]. In particular, weight loss has been shown to slow down knee cartilage degradation [72] and reduce sCOMP levels in OA patients [74].

One of the genes examined in the present study was *ACAN*. The gene encodes aggrecan, which is believed to be responsible for maintaining the proper structure of the articular cartilage [27], and serves as a major component of the proteoglycan gel that reduces the compressive loads on joint cartilage and intervertebral disks [27,31]. In OA, aggrecan is extensively degraded by enzymes such as ADAMTS4 and ADAMTS5, weakening the cartilage structure. To date, few articles have examined the changes in *ACAN* expression occurring during the development of OA [55,56,61].

In the present study, no statistically significant difference in *ACAN* mRNA expression was observed according to OA stage in cartilage. Also, the *ACAN* expression in the Cartilage from the more severely OA-affected medial compartment correlated with its expression in the adjacent cartilage from the relatively preserved lateral compartment. Similar findings were reported by Hashimoto et al. [55], who observed higher *ACAN* expression in advanced OA compared to controls. However, unlike the present study, that analysis was based on gene expression in the hip articular cartilage, and compared diseased cartilage with unaffected control samples collected from different patients.

In contrast to our findings, Zhu et al. report lower *ACAN* mRNA expression in chondrocytes from OA patients compared to controls [56]. The discrepancy may stem from key methodological differences: the present study compared OA-affected and cartilage from the relatively preserved lateral compartment from the same joint, whereas Zhu et al. compared OA samples with healthy cartilage from an independent group of patients experiencing post-traumatic amputations. Furthermore, unlike Zhu et al., our study evaluated a larger patient cohort (*n* = 31 vs. *n* = 20) and comprehensively accounted for demographic factors.

Another novelty of the present study was that unlike most previous studies, it examined changes in *ACAN* mRNA expression in relation to different stages of OA progression.

Due to the influence of external factors and the strong functional and metabolic interactions between cartilage, synovium and subchondral bone, *ACAN* mRNA and protein expression may vary among OA patients. Interestingly, a number of dietary compounds are believed to have chondroprotective effects acting by *inter alia*, thus inhibiting the action of enzymes associated with the breakdown of cartilage components and supporting the production of articular cartilage components [77,78,79,80,81]. In the present study, the participants had undergone knee replacement surgery, and were under medical management of a physician. However, adjuvant therapies were rarely reported: only two patients had received intra-articular hyaluronic acid, while four reported taking oral collagen on an irregular basis.

No statistically significant difference in *COL2A1* mRNA expression was observed according to OA stage in cartilage. This gene encodes type II collagen, a major structural protein of articular cartilage alongside aggrecan [27,31]. Its expression in Cartilage from the more severely OA-affected medial compartment was not correlated with paired cartilage from the relatively preserved lateral compartment. Similarly, Miosge et al. reported a slight increase in type II collagen in stage 3 OA compared to stage 1 OA [38], although this finding was based on a different histopathological classification. The authors suggest that this upregulation reflects a compensatory chondrocyte response to maintain extracellular matrix homeostasis; however, their study lacked a comparison between OA tissues with healthy control cartilage. *COL2A1* expression in cartilage may be influenced by a range of molecular mechanisms; one such possibility involves the use of non-coding RNAs, which may increase the expression of *COL2A1* in chondrocytes [82,83].

In contrast, Zhong et al. reported that *COL2A1* expression gradually decreases with OA progression and negatively correlates with disease severity [54]. Unlike the present study, the analysis was based on Osteoarthritis Research Society International (OARSI) guidelines, and included a smaller patient cohort (*n* = 12 vs. *n* = 31); also, unlike the present study, it compared diseased samples from different individuals with healthy controls. Nevertheless, both studies involved participants with a similar mean age (68 ± 6 vs. 66 ± 7 years). Similarly, Zhu et al. also reported lower levels of *COL2A1* mRNA expression in chondrocytes from OA patients compared with healthy cells [56]. Again, their data were obtained from cartilage samples collected from different participants, with OA and without. Zhu et al. also used different group sizes, and omitted reporting the mean age, sex distribution, mean body weight or OA stage of their cohort [56]. Furthermore, while the authors also reported that OA chondrocytes induced with interleukin-1β (IL-1β) in vitro demonstrated lower type II collagen protein levels compared to controls, this analysis was not performed in our investigation.

Our findings also indicate a significant increase in *COL1A1* mRNA expression with OA progression (stages 3–4), with no correlation observed between OA-affected and cartilage from the relatively preserved lateral compartment. The gene encodes type I collagen, which is typically scarce in healthy chondrocytes. Similar increases have been reported in previous studies [38,54,60]; these alterations have been attributed to extracellular matrix remodeling and chondrocyte phenotype changes during degeneration. Specifically, Chabronova et al. [60] attributed this upregulation to ribosomal RNA methylation; while Zhong et al. [54] also reported that *COL1A1* expression increased with OA advancement, despite using different OA staging methods and smaller cohorts.

Similarly, Miosge et al. reported higher *COL1A1* mRNA expression in late-stage OA cartilage compared to early-stage OA. Although based on the Collins and McElligott (1960) OA stage classification the authors concluded that increased expression of *COL1A1* mRNA in OA tissues is one of the mechanisms of OA pathogenesis [38].

All studied genes demonstrated different levels of expression between the sexes; in particular, *COL2A1* and *ACAN* expression levels were higher in the Cartilage from the more severely OA-affected medial compartment of men with advanced disease (OA stage 3 + 4) compared to women with equivalent disease severity (OA stage 3 + 4). This suggests than men may maintain more effective anabolic processes in the articular cartilage during the course of OA progression.

Together with the degradation of articular cartilage, OA is also characterized by synovial inflammation, driven by *inter alia* mitochondrial dysfunction, and the presence of metabolites, cytokines, and cartilage degradation products in the synovium. These factors can lead to the activation of ependymal cells [84,85].

Although the synovium has been the subject of considerable research, few studies have examined the effect of *ACAN*, *COL2A1*, and *COL1A1* mRNA expression in the synovium during the development of OA. Our present findings confirm a significant increase in *ACAN* mRNA expression in the synovium during OA progression (i.e., at stages 3 + 4) compared to synovial tissue from the relatively preserved lateral compartment. Also, a significant correlation was noted between *ACAN* mRNA expression in OA-affected and synovial tissue from the relatively preserved lateral compartment. An in vitro study by Fahy et al. [61] suggests that *ACAN* expression may be influenced by macrophage polarization, with higher expression levels associated with anti-inflammatory and regenerative processes, possibly mediated by the M1 macrophages present in Synovial tissue from the more severely OA-affected medial compartment.

Our data also indicates a non-significant decrease in *COL2A1* mRNA expression in the synovium during OA development (stages 3–4) compared to synovial tissue from the relatively preserved lateral compartment, with expression levels correlating between OA-affected and synovial tissue from the relatively preserved lateral compartment. Similarly, Fahy et al. [61] reported reduced *COL2A1* expression in mesenchymal stem cells (MSC)-derived cartilage cells exposed to M1 macrophages.

In addition, a non-significant decrease in *COL1A1* mRNA expression was noted at OA stage 3 compared to synovial tissue from the relatively preserved lateral compartment. No significant differences were found between stage 4 and combined stages 3 + 4, and no correlation was observed between OA-affected and synovial tissue from the relatively preserved lateral compartment. Although no significant changes in *COL1A1* expression were observed during OA development in our cohort, other studies reported its upregulation in OA synovium [62,86]. Furthermore, a bioinformatic analysis of GEO data identified *COL1A1* as a potential discriminating factor that increase with knee OA progression [62]. This discrepancy may stem from the smaller sample size of our study; therefore, further studies with larger cohort are needed to evaluate its potential as a biomarker.

Regarding sex differences, lower *ACAN* and *COL2A1* mRNA expressions were observed in the synovial tissue from the more severely OA-affected medial compartment of men (stage 3 + 4) compared to the affected synovium of women with equivalent disease severity (stage 3 + 4). No statistically significant sex-related difference was observed for *COL1A1* mRNA expression.

While the relationship between metabolic comorbidities and OA has been examined previously, available literature remains inconclusive, with some studies indicating a positive association [87,88] and others reporting no relationship [89,90]. Therefore, the present study comprehensively accounted for the presence of metabolic comorbidities, *viz.* diabetes mellitus and hypertension, in our cohort evaluation.

Regarding metabolic comorbidities, no statistically significant differences in synovial *ACAN*, *COL1A1*, or *COL2A1* mRNA expression were observed between patients with and without diabetes or hypertension. Given the small subgroup sizes, these findings should be interpreted cautiously and require confirmation in larger cohorts. Interestingly, in hypertensive patients, significantly higher *COL1A1* mRNA expression was noted in Cartilage from the more severely OA-affected medial compartment compared to cartilage from the relatively preserved lateral compartment. A considerable body of research exists on the influence of diabetes on the occurrence of OA. A systematic literature review and meta-analysis by Louati et al. found that patients with diabetes mellitus (DM) had a 1.46-fold higher risk (OR = 1.46) of developing OA compared to patients without DM [87]. Furthermore, studies indicate that patients with DM tend to demonstrate more severe OA symptoms, as measured by the Knee Injury and Osteoarthritis Outcome Score (KOOS) and the Western Ontario and McMaster Universities Arthritis Index (WOMAC), compared to those without DM [91]. Moreover, patients with OA and DM have also been found to be more at risk of synovitis [91]. Eymard et al. reported a more rapid progression of joint space narrowing in the medial tibiofemoral compartment in patients with knee OA and type 2 diabetes than those with OA alone [92]. However, a meta-analysis by Khor et al. did not confirm DM an independent risk factor for OA, and population-based studies by Frey et al. did not identify any relationship between non-insulin-dependent diabetes mellitus (NIDDM) and OA [89,93]. Also, Funck-Brentano et al. did not identify a causal relationship between type II diabetes and knee OA [94].

One mechanism that may explain the increased risk of OA in patients with DM is the induction of low-grade systemic inflammation by hyperglycemia [95,96]. Inflammation can cause cartilage damage, and hence is a driver of OA pathogenesis [97]. In addition, patients with DM also exhibit increased production of mitochondrial [98] and cytosolic ROS [99]; promoting the risk of cartilage damage through chondrocyte apoptosis [100], disruption of cartilage proteoglycan synthesis in response to IL-1 [101], and the activation of metal-dependent proteases [102]. Furthermore, healthy chondrocytes, i.e., without OA, regulate the synthesis and degradation of glucose transporters such as Glucose Transporter Type 1 (GLUT-1), allowing them to adjust intracellular glucose concentrations relative to extracellular levels [91]. However, chondrocytes with OA lose this potential when exposed to high glucose concentrations, leading to increased intracellular glucose accumulation and increased ROS production [103]. Another research has also found advanced glycation end products (AGEs) to accumulate in cartilage, resulting in increased matrix stiffness and decreased resistance to mechanical stress [104].

Hypertension has also been widely studied for its impact on the incidence and progression of OA. A systematic review and meta-analysis of observational studies found that hypertension increases the risk of knee OA by as much as 62% [88]. The study also highlighted an association with structural joint damage, linking hypertension to an increased risk of radiographic OA (89%) and symptomatic OA (39%). However, because the included studies were mostly cross-sectional, no causal relationship could be confirmed [88]. Conversely, a Korean population-based study demonstrated an inverse relationship between hypertension and the incidence of OA, with OA being less frequent in patients with systolic blood pressure ≥140 mmHg compared to those with systolic blood pressure <120 mmHg [90]. Funck-Brentano et al. also reported that systolic blood pressure was inversely associated with knee OA [94].

The increased risk of OA associated with hypertension may be explained by the influence of blood flow on the osteochondral system. De Lorenzo et al. reported a positive correlation between systemic blood pressure and intra-osseous pressure in long bones in a swine model [105]. Abnormal blood flow through the bone tissue results in osteocyte apoptosis, triggering subchondral bone resorption [106], which in turn may lead to the development of OA [107].

During the development of OA, aggrecan and type II collagen are progressively degraded. Although no statistically significant differences in *ACAN* or *COL2A1* mRNA expression were observed in cartilage, ACAN mRNA expression was significantly higher in synovial tissue from the more severely OA-affected medial compartment.

The analytical methods employed in the present study are commonly used in molecular assessment in OA research [46,108,109,110,111]. RNA isolation from cartilage and synovium was performed using the column-based method, which is considered more efficient and yields higher RNA purity than classical phenol/chloroform methods for RNA isolation from cartilage and synovium [108]. Specifically, RNA was isolated using the mirVana Isolation Kit, a well-documented approach in OA studies that reliably yields high-purity RNA [109,110,112]. Total RNA concentration was determined by spectrophotometry, consistent with standard protocols in cartilage studies [111].

The relative expression (RQ) of the studied genes was assessed by real-time PCR: an efficient, accurate [45], and reliable method for measuring gene expression [113], commonly employed in studies on OA [46]. However, a key consideration in RT-PCR assays is the selection of an appropriate reference gene. In the present study the hypoxanthine-guanine phosphoribosyltransferase (*HPRT1*) gene was selected as the endogenous control; as its expression stability has been previously validated in both cartilage [45] and synovium [46]. Indeed, *HPRT1* has been previously identified as the most suitable reference gene for PCR standardization [45,46].

A protein–protein interaction network prepared using the STRING web platform found ACAN, COL2A1, and COL1A1 to be associated with various proteins involved in cartilage and synovial processes. More specifically, the studied proteins were found to be associated with HAPLN1: a protein that strongly interacts with ACAN and COL2A1, but weakly with COL1A1. The main function of HAPLN1 is to stabilize ECM components [114,115], thereby increasing resistance to extracellular matrix compression and improving joint cushioning [116]. Zheng et al. suggested that the HAPLN1 dysregulation may play a key role in the pathogenesis and structural breakdown of osteoarthritis [116].

Furthermore, ACAN was found to interact strongly with PRG4, also known as lubricin, while COL2A1 and COL1A1 demonstrated weak interactions. PRG4 has lubricating properties and is necessary for maintaining cartilage hydration and tissue homeostasis; it is expressed in the articular cartilage and synovium [117].

Bioinformatic analysis also found ACAN, COL2A1, and COL1A1 to be associated with ADAMTS3, ADAMTS4 and ADAMTS5. Specifically, ADAMTS3 and ADAMTS4 strongly interact with ACAN and COL2A1, while showing moderate association with COL1A1. ADAMTS5, on the other hand, strongly interacts with ACAN and COL2A1, but weakly with COL1A1. The expression of the metalloproteinases ADAMTS-3 (also called procollagen N-peptidase), ADAMTS-4 (also called aggrecanase-1), and ADAMTS-5 (also called aggrecanase-2) [31] were also found to increase in response to elevated level of inflammatory factors associated with OA development [118].

ADAMTS-4 and ADAMTS-5 are responsible for the cleavage of the Glu373-Ala374 bond in aggrecan, indicating their participation in cartilage degradation [31]. ADAMTS-3 is involved in the degradation of procollagen I, II, III, and V, which results in the formation of specific collagen fibers and, thus, endochondral ossification [31]. Studies on rats found *ADAMTS5* expression to increase with the progression of OA induced by surgery for meniscal tears in the knee joint [119].

A bioinformatic analysis also found ACAN, COL2A1, and COL1A1 to be associated with MATN1. The MATN1 protein interacts strongly with ACAN, moderately with COL2A1, and weakly with COL1A1. Matrilin family proteins are ECM adaptor proteins. They interact with ECM cartilage components (including aggrecan and collagens II and IX), which help stabilize the macromolecular network of collagen fibrils and aggrecan. Although previous studies reported no significant differences in *MATN1* genotype frequencies between OA patients and controls. Ref. [120], functional studies by Li et al. demonstrated that matrilin-knockout mice are significantly more susceptible to age-related OA, indicating that matrilin deficiency contributes directly to joint breakdown [121].

The prepared PPI network also showed that COL11A2 protein interacts strongly with COL2A1 and COL1A1 and weakly with ACAN. Together, COL11A2, COL11A1, and COL11A3 form type XI collagen, which is responsible for regulating the production of collagen fibers by type II collagen [122,123]. One case report indicated that biallelic variants of the *COL11A2* gene *in trans* may be associated with the development of OA [123].

Our present bioinformatic analysis found the levels of ACAN, COL2A1 and COL1A1 proteins to be associated with those of SCX. The level of SCX is regulated by transforming growth factor β (TGF-β), which governs the activation of genes involved in tendon differentiation. One of these is *COL1A1*, encoding the α1 chain of type I collagen [124].

The bioinformatic analysis also found CRTAP to be strongly associated with COL1A1 and moderately so with COL2A1, while no association was demonstrated between CRTAP and ACAN. CRTAP is involved in the formation of collagen fibers in cartilage and bones [125]. It is a component of the collagen prolyl 3-hydroxylation complex in the endoplasmic reticulum, regulating the function of chaperone proteins [126].

A previous analysis identified 27 differentially expressed genes (DEGs) between OA and non-OA cartilage from the National Center for Biotechnology Information database. Among these, five central genes were identified, including *COL1A1* and *COL1A2*, which were involved in the relaxin signaling pathway [127]. Enrichment analysis of all the identified DEGs demonstrated their involvement in functional areas related to *inter alia* collagen fibrils, being structural components of the extracellular matrix (including providing tensile strength), and in platelet-derived growth factor binding [127].

The expression of genes encoding proteins included in the presented PPI network were also analyzed using the Orange Data Mining platform. The datasets consisted of transcriptome data obtained from patient tissues during knee replacement surgery at the Grossman School of Medicine (New York, USA) using the Affymetrix Human Genome U133 Plus 2.0 platform. In particular chondrocytes, higher *ACAN*, *COL1A1*, *ADAMTS3*, *ADAMTS4*, and *HAPLN1* mRNA expression was observed in the late-stage of OA compared to early stage; however, *COL2A1*, *ADAMTS5*, *CRTAP*, and *COL11A2* mRNA expression decreased with disease progression. In parallel, the OA synovium demonstrated increased *COL1A1* and *COL11A1* mRNA expression and decreased *ACAN* and *ADAMTS5* mRNA expression compared to normal synovium.

Bioinformatic analysis based on PPI networks and GO annotation provides a powerful, systems-level framework for interpreting high-throughput transcriptomic data by revealing functional relationships among genes and proteins and identifying key network hubs and signaling pathways By generating statistically supported functional annotations across biological processes, molecular functions, and cellular components, these integrative approaches significantly enhance biological interpretability, prioritize candidate biomarkers for experimental validation, and enable robust hypothesis generation and verification across large-scale omics datasets [50,128,129].

Based on these data, Spearman’s correlation analysis identified a strong positive correlation between chondrocyte *COL1A1* mRNA expression, and the expression of *ACAN*, *PRG4*, *SCX* and *ADAMTS4* mRNA; negative correlations were also noted between *COL1A1* mRNA and *CRTAP* and *ADAMTS5* mRNA expression. Another Spearman’s correlation analysis based on data downloaded from the GEO database identified a negative correlation between *ACAN* and *ADAMTS5* mRNA expression as well as between *COL2A1* and *HAPLN1* mRNA expression, while showing a positive correlation between *COL2A1* and *COL11* mRNA expression. In the synovium, *COL1A1* mRNA expression correlated negatively with *ACAN*, *CRTAP* and *ADAMTS5* mRNA levels.

Limitation: This study was conducted as an observational pilot analysis supported by preliminary bioinformatic interpretation. When interpreting our findings, it is important to consider that they were obtained through a single-center study with a relatively small sample size, which influenced the statistical analysis and test power used in the study.

The relatively small sample size represents an important limitation of the present pilot study. This limitation becomes particularly relevant after stratification according to OA stage, sex, or metabolic comorbidities, resulting in small subgroups and limited statistical power. Therefore, subgroup findings should be regarded as exploratory and hypothesis-generating and require confirmation in larger, prospectively designed cohorts. Statistical significance observed in individual subgroups should not be interpreted as evidence of a differential subgroup effect in the absence of a formal interaction analysis. In addition, because multiple exploratory subgroup and correlation analyses were performed without formal adjustment for multiplicity, an increased risk of type I error cannot be excluded; therefore, nominally significant findings from these analyses should be interpreted as hypothesis-generating and require independent confirmation.

The present study used a hypothesis-driven candidate-gene approach rather than an untargeted omics screening strategy. *ACAN*, *COL1A1*, and *COL2A1* were selected a priori based on their established roles in cartilage extracellular matrix homeostasis and OA-related remodeling. Therefore, the subsequent PPI, GO, and GEO analyses were not intended for target discovery or formal external validation, but rather to provide complementary biological context for the experimental findings. The GEO datasets were used as an external reference to assess whether the observations obtained in our cohort were broadly consistent with expression patterns reported in other OA datasets. However, differences in patient characteristics, tissue sources, disease severity, and analytical platforms between the present cohort and publicly available GEO datasets limit direct comparisons. Therefore, the in silico analyses should be regarded as exploratory and hypothesis-generating rather than as a validation step.

Further experimental validation of these results, especially identified molecular pathways and dysregulated inflammatory mediators, will require expansion of the patient cohort, collection of additional biological samples, and approval from the institutional bioethical committee. These steps are already planned as part of our forthcoming research phase, which will focus on functional and molecular verification of the mechanisms proposed in this study. Differential analysis between the medial and lateral knee compartments is also planned to discover new molecular targets for future research. Therefore, the current work should be regarded as a preliminary integrative investigation establishing a solid foundation for future experimental validation.

To conclude, both joint cartilage and synovium undergo similar changes in *ACAN* mRNA expression during OA progression, but differ with regard to *COL1A1* and *COL2A1* mRNA expression. Osteoarthritis was associated with an increase in *ACAN* mRNA expression in both compartments, although the change achieved statistical significance only in the synovium. This observed change may represent a compensatory mechanism aimed at offsetting damage caused by aggrecan degradation in the joint. Additionally, patients with diabetes demonstrated higher *ACAN* mRNA expression in articular cartilage and synovium than non-diabetic patients and although this difference was statistically non-significant, it still indicates that diabetes may exacerbate the molecular changes associated with OA.

*COL1* and *COL2* mRNA expression varied between cartilage and synovium samples. *COL1A1* expression was significantly higher in Cartilage from the more severely OA-affected medial compartment (stage 3 + 4) compared to cartilage from the relatively preserved lateral compartment. In the exploratory subgroup analysis, significantly higher *COL1A1* mRNA expression in the more severely OA-affected medial cartilage was also observed among patients with hypertension. However, in the absence of a formal interaction analysis, this finding should not be interpreted as evidence that hypertension modifies the compartment-specific difference in *COL1A1* expression. In the synovial membranes, type I collagen is responsible for synovial fibrosis in OA. Our data indicated that *COL1A1* expression was statistically nonsignificant lower in stage 3 of disease progression, but statistically nonsignificant higher in stage 4, with the latter reaching levels comparable to control tissues. The lack of a marked increase in *COL1A1* mRNA expression in OA-affected synovial membranes may be due to the concomitant observed increase in *ACAN* mRNA expression, which could provide a compensatory protective mechanism by increased aggrecan level against matrix breakdown.

*COL2A1* mRNA expression in cartilage from the more severely OA-affected medial compartment was found to differ from cartilage from the relatively preserved lateral compartment. Interestingly, while a statistical non-significant decrease was noted in stage 3 Cartilage from the more severely OA-affected medial compartment, a considerable increase was observed in OA-affected (stage 4) cartilage, though, neither change reached statistical significance. In the synovial membranes, a statistical non-significant decrease in *COL2A1* mRNA expression was observed in OA patients, which is characteristic of disease progression. While cartilage and synovium differed with regard to *COL1A1* and *COL2A1* expression, both tissues demonstrated a statistical non-significant higher expression of both genes in OA-affected stage 4 compared to OA-affected stage 3. Furthermore, the *COL2A1* expression in Cartilage from the more severely OA-affected medial compartment and synovium was statistically non-significantly higher in patients with hypertension compared to those without.

## 5. Conclusions

Osteoarthritis development is a multi-stage, dynamic process resulting in progressive joint degradation accompanied by compensatory molecular mechanisms.

Our findings indicate that *ACAN* expression follows a similar direction in both articular cartilage and synovium, while *COL1A1* and *COL2A1* expression patterns diverge between tissue types.

Exploratory subgroup analyses suggested that metabolic comorbidities may be associated with variation in gene-expression patterns; however, these observations require confirmation in larger cohorts. This was to be expected, as gene expression is influenced by strong functional and metabolic interactions between cartilage, synovium, and subchondral bone.

We believe that our findings may act as a solid foundation for further research into molecular markers for monitoring OA progression. Moreover, identifying compartment-specific molecular heterogeneity within a single joint may support clinical decision-making regarding surgical interventions, such as favoring unicompartmental arthroplasty over total joint replacement, ultimately enhancing patient quality of life and accelerating postoperative rehabilitation.

## Figures and Tables

**Table 1:** Clinical characteristics of the included OA patients.

Variable	Mean	Median	SD
age [years]	66	68	7
height [m]	1.65	1.64	0.08
weight [kg]	90.42	88	12.12
BMI [kg/m^2^]	33.03	32.85	3.12

BMI—body mass index.

**Table 2:** Relative expression (RQ) of genes in relatively preserved lateral and OA-affected articular cartilage.

Gene Name	OA Grading	Median RQ	IQR
*ACAN*	Relatively preserved lateral	226.53	105.87–874.62
	OA-affected medial (stage 3)	260.06	48.37–478.89
	OA-affected medial (stage 4)	730.67	153.09–1312.69
	OA-affected medial (stage 3 + 4)	291.26	151.42–936.03
*COL1A1*	Relatively preserved lateral	1.06	0.59–3.45
	OA-affected medial (stage 3)	3.55	1.08–10.19
	OA-affected medial (stage 4)	5.27 *	1.70–7.39
	OA-affected medial (stage 3 + 4)	5.04 *	1.34–1.63
*COL2A1*	Relatively preserved lateral	57.07	20.25–311.33
	OA-affected medial (stage 3)	52.84	25.09–319.87
	OA-affected medial (stage 4)	215.36	57.20–371.06
	OA-affected medial (stage 3 + 4)	132.03	28.16–371.06

Sample sizes for individual variants: Relatively preserved lateral (*n* = 31); stage 3 (*n* = 14); stage 4 (*n* = 17); OA-affected (stage 3 + 4) (*n* = 31). IQR—Interquartile range. Significance: *—*p* < 0.05. For the six prespecified primary comparisons (three genes in articular cartilage and three genes in synovial tissue), multiplicity was controlled using the Holm procedure.

**Table 3:** Correlation between the expression of the tested genes (RQ) with OA stage in articular cartilage.

Gene Name	OA Grading	Correlation Between Gene Expression and OA Severity Spearman Correlation Coefficients
*ACAN*	Relatively preserved lateral vs. OA-affected medial (stage 3)	0.121
	Relatively preserved lateral vs. OA-affected medial (stage 4)	0.517 *
	Relatively preserved lateral vs. OA-affected medial (stage 3 + 4)	0.396 *
*COL1A1*	Relatively preserved lateral vs. OA-affected medial (stage 3)	0.310
	Relatively preserved lateral vs. OA-affected medial (stage 4)	−0.069
	Relatively preserved lateral vs. OA-affected medial (stage 3 + 4)	0.040
*COL2A1*	Relatively preserved lateral vs. OA-affected medial (stage 3)	0.024
	Relatively preserved lateral vs. OA-affected medial (stage 4)	0.186
	Relatively preserved lateral vs. OA-affected medial (stage 3 + 4)	0.127

Significance: *—*p* < 0.05.

**Table 4:** Expression level (RQ) of study genes in unaffected and OA-affected articular cartilage.

Sex	Gene Name	Variant	Median RQ	IQR
Female	*ACAN*	Relatively preserved lateral	221.71	105.87–874.62
		OA-affected medial (stage 3 + 4)	288.28	82.46–535.29
	*COL1A1*	Relatively preserved lateral	1.07	0.71–3.45
		OA-affected medial (stage 3 + 4)	5.04	1.34–7.63
	*COL2A1*	Relatively preserved lateral	79.03	23.59–366.53
		OA-affected medial (stage 3 + 4)	79.04	25.09–319.88
Male	*ACAN*	Relatively preserved lateral	229.01	124.13–666.73
		OA-affected medial (stage 3 + 4)	755.55	212.77–1312.69
	*COL1A1*	Relatively preserved lateral	0.94	0.59–2.29
		OA-affected medial (stage 3 + 4)	5.85 *	1.70–7.39
	*COL2A1*	Relatively preserved lateral	31.23	10.97–231.44
		OA-affected medial (stage 3 + 4)	179.67	50.77–371.06

Sample sizes for individual variants: female cartilage from the relatively preserved lateral compartment (*n* = 21); female Cartilage from the more severely OA-affected medial compartment (stage 3 + 4) (*n* = 21); male cartilage from the relatively preserved lateral compartment (*n* = 10); male cartilage from the more severely OA-affected medial compartment (stage 3 + 4) (*n* = 10). IQR—Interquartile range. Significance: *—*p* < 0.05.

**Table 5:** Correlation in tested gene expression between OA-affected medial compartment and cartilage from the relatively preserved lateral compartment by sex.

Sex	Gene Name	Variant	Correlation Between Gene Expression and OA Severity Correlation Coefficients
Female	*ACAN*	Relatively preserved lateral vs. OA-affected medial (stage 3 + 4)	0.347
	*COL1A1*	Relatively preserved lateral vs. OA-affected medial(stage 3 + 4)	0.110
	*COL2A1*	Relatively preserved lateral vs. OA-affected medial (stage 3 + 4)	0.121
Male	*ACAN*	Relatively preserved lateral vs. OA-affected medial (stage 3 + 4)	0.430
	*COL1A1*	Relatively preserved lateral vs. OA-affected medial (stage 3 + 4)	−0.133
	*COL2A1*	Relatively preserved lateral vs. OA-affected medial (stage 3 + 4)	0.164

**Table 6:** Expression level (RQ) of study genes in relatively preserved lateral compartment and OA-affected medial compartment articular cartilage.

Gene Name	Variant OA	Comorbidity	Median RQ	IQR
*ACAN*	Relatively preserved lateral	diabetes	197.61	84.80–550.58
		no diabetes	231.50	114.17–666.73
	OA-affected medial (stage 3 + 4)	diabetes	297.02	182.60–793.90
		no diabetes	291.26	146.71–780.42
*COL1A1*	Relatively preserved lateral	diabetes	0.49	0.26–2.21
		no diabetes	1.38	0.94–4.63
	OA-affected medial (stage 3 + 4)	diabetes	5.47	2.67–8.79
		no diabetes	5.00	1.43–13.32
*COL2A1*	Relatively preserved lateral	diabetes	218.56	34.02–2030.07
		no diabetes	47.60	17.33–190.15
	OA-affected medial (stage 3 + 4)	diabetes	138.00	37.93–252.86
		no diabetes	84.21	28.16–371.06
*ACAN*	Relatively preserved lateral	hypertension	203.13	114.95–377.17
		no hypertension	231.50	96.45–666.73
	OA-affected medial (stage 3 + 4)	hypertension	289.77	152.42–673.60
		no hypertension	304.90	146.71–936.03
*COL1A1*	Relatively preserved lateral	hypertension	0.94	0.39–1.07
		no hypertension	2.61	1.38–4.63
	OA-affected medial (stage 3 + 4)	hypertension	6.32 *	2.92–12.65
		no hypertension	4.35	0.82–6.70
*COL2A1*	Relatively preserved lateral	hypertension	102.41	21.00–311.33
		no hypertension	47.60	14.36–231.44
	OA-affected medial (stage 3 + 4)	hypertension	183.15	28.45–319.87
		no hypertension	84.21	26.84–287.46

Sample sizes for individual variants: Diabetes with relatively preserved lateral compartment (*n* = 8); Diabetes with OA-affected medial compartment (stage 3 + 4) (*n* = 8); No diabetes with relatively preserved lateral compartment (*n* = 19); No diabetes with OA-affected medial compartment (stage 3 + 4) (*n* = 19); Hypertension with relatively preserved lateral compartment (*n* = 14); Hypertension with OA-affected medial compartment (stage 3 + 4) (*n* = 14); No hypertension with relatively preserved lateral compartment (*n* = 13); No hypertension with OA-affected medial compartment (stage 3 + 4) (*n* = 13). IQR—Interquartile range. Significance: *—*p* < 0.05.

**Table 7:** Relative expression (RQ) of the tested genes in OA-affected medial compartment and synovial tissue from the relatively preserved lateral compartment.

Gene Name	OA Grading	Median RQ	IQR
*ACAN*	Relatively preserved lateral	0.004	0.002–0.017
	OA-affected medial (stage 3)	0.02	0.003–0.048
	OA-affected medial (stage 4)	0.02	0.002–0.055
	OA-affected medial (stage 3 + 4)	0.02 *	0.002–0.055
*COL1A1*	Relatively preserved lateral	0.02	0.011–0.050
	OA-affected medial (stage 3)	0.01	0.001–0.101
	OA-affected medial (stage 4)	0.02	0.004–0.049
	OA-affected medial (stage 3 + 4)	0.02	0.003–0.101
*COL2A1*	Relatively preserved lateral	6.94	0.87–23.91
	OA-affected medial (stage 3)	3.56	0.41–7.77
	OA-affected medial (stage 4)	4.05	0.71–33.03
	OA-affected medial (stage 3 + 4)	3.88	0.64–33.03

Sample sizes for individual variants: Relatively preserved lateral (*n* = 31); stage 3 (*n* = 14); stage 4 (*n* = 17); OA-affected medial (stage 3 + 4) (*n* = 31). IQR—Interquartile range. Significance: *—*p* < 0.05.

**Table 8:** Correlation between the expression of the tested genes (RQ) with OA stages in the synovium.

Gene Name	OA Grading	Correlation Between Gene Expression and OA Severity Spearman’s Correlation Coefficients
*ACAN*	Relatively preserved lateral vs. OA-affected medial (stage 3)	0.696 *
	Relatively preserved lateral vs. OA-affected medial (stage 4)	0.350
	Relatively preserved lateral vs. OA-affected medial (stage 3 + 4)	0.464 *
*COL1A1*	Relatively preserved lateral vs. OA-affected medial (stage 3)	−0.154
	Relatively preserved lateral vs. OA-affected medial (stage 4)	0.034
	Relatively preserved lateral vs. OA-affected medial (stage 3 + 4)	−0.082
*COL2A1*	Relatively preserved lateral vs. OA-affected medial (stage 3)	0.675 *
	Relatively preserved lateral vs. OA-affected medial (stage 4)	0.551 *
	Relatively preserved lateral vs. OA-affected medial (stage 3 + 4)	0.634 *

Significance: *—*p* < 0.05.

**Table 9:** Expression level (RQ) of genes in relatively preserved lateral compartment and synovial tissue from the more severely OA-affected medial compartment.

Sex	Gene Name	Variant (n)	Median RQ	IQR
Female	*ACAN*	Relatively preserved lateral	0.003	0.001–0.008
		OA-affected medial (stage 3 + 4)	0.023 *	0.006–0.055
	*COL1A1*	Relatively preserved lateral	0.018	0.009–0.051
		OA-affected medial (stage 3 + 4)	0.016	0.003–0.059
	*COL2A1*	Relatively preserved lateral	8.251	1.594–23.915
		OA-affected medial (stage 3 + 4)	4.361	1.227–33.028
Male	*ACAN*	Relatively preserved lateral	0.009	0.004–0.017
		OA-affected medial (stage 3 + 4)	0.014	0.001–0.042
	*COL1A1*	Relatively preserved lateral	0.028	0.015–0.042
		OA-affected medial (stage 3 + 4)	0.019	0.001–0.137
	*COL2A1*	Relatively preserved lateral	4.670	0.128–8.186
		OA-affected medial (stage 3 + 4)	0.799	0.164–20.366

Sample sizes for individual variants: female synovial tissue from the relatively preserved lateral compartment (*n* = 21); female synovial tissue from the more severely OA-affected medial compartment (stage 3 + 4) (*n* = 21); male synovial tissue from the relatively preserved lateral compartment (*n* = 10); male synovial tissue from the more severely OA-affected medial compartment (stage 3 + 4) (*n* = 10). IQR—Interquartile range. Significance: *—*p* < 0.05.

**Table 10:** Correlation of gene expression between OA-affected and synovial tissue from the relatively preserved lateral compartment with regard to sex.

Sex	Gene Name	Variant (n)	Correlation Between Gene Expression and OA Severity Correlation Coefficients
Female	*ACAN*	Relatively preserved lateral vs. OA-affected medial (stage 3 + 4)	0.600 *
	*COL1A1*	Relatively preserved lateral vs. OA-affected medial (stage 3 + 4)	−0.009
	*COL2A1*	Relatively preserved lateral vs. OA-affected medial (stage 3 + 4)	0.400 *
Male	*ACAN*	Relatively preserved lateral vs. OA-affected medial (stage 3 + 4)	0.164
	*COL1A1*	Relatively preserved lateral vs. OA-affected medial (stage 3 + 4)	−0.248
	*COL2A1*	Relatively preserved lateral vs. OA-affected medial (stage 3 + 4)	0.900 *

Significance: *—*p* < 0.05.

## Data Availability

Dataset available on request from the authors.

## Supplementary Materials

The following supporting information can be downloaded at: https://www.mdpi.com/article/10.3390/jcm15207977/s1: Figure S1. Spearman’s correlation matrix of the chosen genes’ mRNA expression in chondrocytes. Chondrocytes isolated from late and early OA-affected knee joints. Transcriptomic data were downloaded from the GSE179716 dataset of the GEO database. Analysis and visualization were performed using JASP 0.16.0.0 software. Spearman’s ρ value is depicted numerically and as color intensity. * *p* > 0.05, ** *p* > 0.005, and *** *p* > 0.001; Figure S2. Spearman’s correlation matrix of the chosen genes’ mRNA expression in synovium. Synovium isolated from normal and OA-affected knee joints. Transcriptomic data were downloaded from the GSE206848 dataset of the GEO database. Analysis and visualization were performed using JASP 0.16.0.0 software. Spearman’s ρ value is depicted numerically and as color intensity. * *p* > 0.05, ** *p* > 0.005, and *** *p* > 0.001.

## Abbreviations

The following abbreviations are used in this manuscript:

ACAN	aggrecan
ADAMTS3	A Disintegrin and Metalloproteinases with Thrombospondin motifs type 3; procollagen N-peptidase
ADAMTS4	A Disintegrin and Metalloproteinases with Thrombospondin motifs type 4; aggrecanase-1
ADAMTS5	A Disintegrin and Metalloproteinases with Thrombospondin motifs type 5; aggrecanase-2
AGEs	Advanced Glycation End Products
BMI	body mass index
BP	biological process
cDNA	complementary DNA
COL1A1	collagen type I
COL2A1	collagen type II
COL11A2	collagen type XI
CRTAP	cartilage-related protein
CT	threshold cycle
CTX-II	C-terminal telopeptide of type II collagen
DAMPs	damage-associated molecular patterns
DEGs	differentially expressed genes
dNTP	deoxyribonucleotide triphosphates
ECM	extracellular matrix
GEO	The Gene Expression Omnibus
GLUT-1	Glucose Transporter Type 1
GO	Gene Ontology
HAPLN1	hyaluronan-proteoglycan-linking protein 1
HPRT1	hypoxanthine-guanine phosphoribosyltransferase
IFP	infrapatellar fat pads
IL-1β	interleukin-1β
IQR	Interquartile range
KOOS	Knee Injury and Osteoarthritis Outcome Score
MATN1	matrilin-1
MF	molecular functions
MPC	mesenchymal progenitor cell
MSC	mesenchymal stem cells
NIDDM	non-insulin-dependent diabetes mellitus
NF-kB	nuclear factor-kB
OA	osteoarthritis
OARSI	Osteoarthritis Research Society International
PCR	polymerase chain reaction
PPI	Protein–Protein Interaction
PRG4	proteoglycan 4
rho	Spearman’s rank correlation coefficient
RI	Rnase inhibitor
ROS	Reactive oxygen species
RT	reverse transcription
RQ	relative expression
sCOMP	Serum Cartilage Oligomeric Matrix Protein
SCX	transcription factor scleraxis
TGF-β	Transforming Growth Factor β
WOMAC	Western Ontario and McMaster Universities Arthritis Index
