# Supplementary material for: Differential Expression of ACAN, COL1A1, and COL2A1 in Cartilage and Synovium During Osteoarthritis Progression: An Observational Pilot Study Supported by Preliminary Bioinformatic Analysis
Source: J Clin Med. 2026 Oct 15;15(20):7977. doi: 10.3390/jcm15207977 (PMC13706629; doi:10.3390/jcm15207977)
Supplement: Supplementary file 1 [file jcm-15-07977-s001.zip › jcm-4502059-supplementary.pdf]

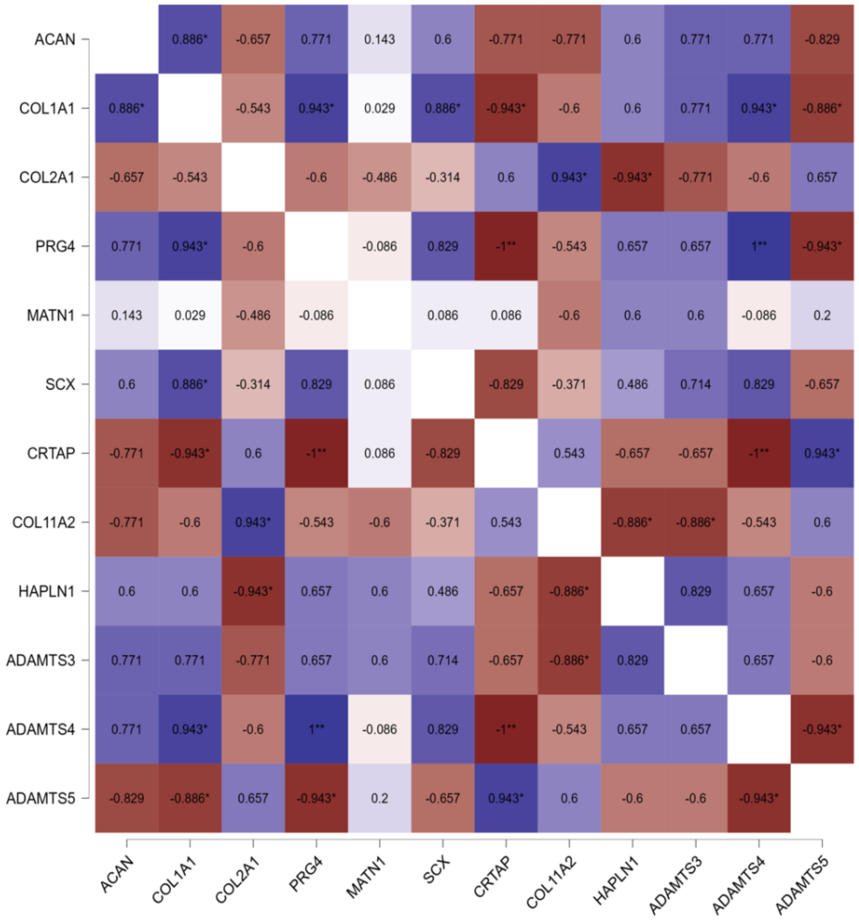

Figure S1. Spearman's correlation matrix of the chosen genes' mRNA expression in chondrocytes. Chondrocytes isolated from late and early OA-affected knee joints. Transcriptomic data were downloaded from the GSE179716 dataset of the GEO database. Analysis and visualization were performed using JASP 0.16.0.0 software. Spearman's ρ value is depicted numerically and as color intensity. * $p > 0.05$, ** $p > 0.005$, and *** $p > 0.001$

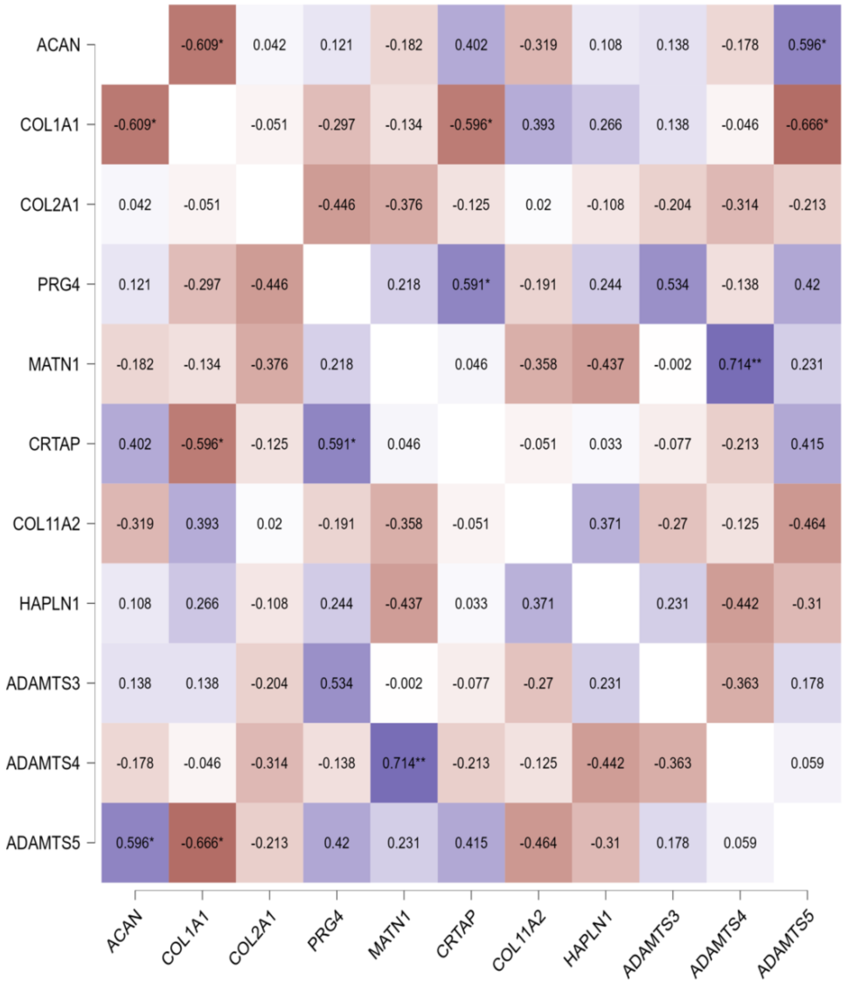

Figure S2. Spearman's correlation matrix of the chosen genes' mRNA expression in synovium. Synovium isolated from normal and OA-affected knee joints. Transcriptomic data were downloaded from the GSE206848 dataset of the GEO database. Analysis and visualization were performed using JASP 0.16.0.0 software. Spearman's ρ value is depicted numerically and as color intensity. * $p > 0.05$, ** $p > 0.005$, and *** $p > 0.001$
